# Effectiveness of Non-Pharmacological Interventions to Prevent Falls in Older People: A Systematic Overview. The SENATOR Project ONTOP Series

**DOI:** 10.1371/journal.pone.0161579

**Published:** 2016-08-25

**Authors:** Joseph M. Rimland, Iosief Abraha, Giuseppina Dell’Aquila, Alfonso Cruz-Jentoft, Roy Soiza, Adalsteinn Gudmusson, Mirko Petrovic, Denis O’Mahony, Chris Todd, Antonio Cherubini

**Affiliations:** 1 Geriatrics and Geriatric Emergency Care, Italian National Research Center on Aging, Ancona, Italy; 2 Division of Geriatrics, Hospital Universitario Ramón y Cajal, Madrid, Spain; 3 Department of Medicine for the Elderly, Woodend Hospital, Aberdeen, United Kingdom; 4 Landspitali University Hospital Reykjavik, Reykjavik, Iceland; 5 Ghent University Hospital, Ghent, Belgium; 6 Division of Geriatrics, Department of Medicine, University College Cork, Cork, Ireland; 7 School of Health Sciences, The University of Manchester, Manchester, United Kingdom; Universidade Federal do Rio de Janeiro, BRAZIL

## Abstract

**Background:**

Falls are common events in older people, which cause considerable morbidity and mortality. Non-pharmacological interventions are an important approach to prevent falls. There are a large number of systematic reviews of non-pharmacological interventions, whose evidence needs to be synthesized in order to facilitate evidence-based clinical decision making.

**Objectives:**

To systematically examine reviews and meta-analyses that evaluated non-pharmacological interventions to prevent falls in older adults in the community, care facilities and hospitals.

**Methods:**

We searched the electronic databases Pubmed, the Cochrane Database of Systematic Reviews, EMBASE, CINAHL, PsycINFO, PEDRO and TRIP from January 2009 to March 2015, for systematic reviews that included at least one comparative study, evaluating any non-pharmacological intervention, to prevent falls amongst older adults. The quality of the reviews was assessed using AMSTAR and ProFaNE taxonomy was used to organize the interventions.

**Results:**

Fifty-nine systematic reviews were identified which consisted of single, multiple and multifactorial non-pharmacological interventions to prevent falls in older people. The most frequent ProFaNE defined interventions were exercises either alone or combined with other interventions, followed by environment/assistive technology interventions comprising environmental modifications, assistive and protective aids, staff education and vision assessment/correction. Knowledge was the third principle class of interventions as patient education. Exercise and multifactorial interventions were the most effective treatments to reduce falls in older adults, although not all types of exercise were equally effective in all subjects and in all settings. Effective exercise programs combined balance and strength training. Reviews with a higher AMSTAR score were more likely to contain more primary studies, to be updated and to perform meta-analysis.

**Conclusions:**

The aim of this overview of reviews of non-pharmacological interventions to prevent falls in older people in different settings, is to support clinicians and other healthcare workers with clinical decision-making by providing a comprehensive perspective of findings.

## Introduction

Falls are common in older people, with one in three older people falling at least once during a year [1]. Moreover, the propensity of older adults to fall increases with age, more than doubling between 70 and 80 years of age [2]. Fall-related injuries also rise with age, with an increased risk of fracture, which in more than half of the cases occur to the hip [3]. In addition to injuries, falling can also induce fear of falling, which leads to further falling, avoiding or restricting daily activities, losing autonomy, diminishing social activity, depression and deterioration of quality of life [4, 5]. Fear of falling in older people can be measured with the Falls Efficacy Scale-International [6]. Fall risk factors include a history of falls, gait problems, walking aid use, vertigo, muscle weakness [7], a variety of drugs, particularly psychotropic drugs [8] and polypharmacy [9]. Fall prevention programs targeting various risk factors have been carried out with older people living at home, in care facilities (e.g., long-term, residential, nursing homes) and in hospitals (acute and sub-acute wards) and consist of single, multiple and multifactorial interventions.

Systematic reviews (SRs) of clinical trials are very useful for healthcare professionals, researchers, and policymakers, by identifying and summarizing a vast amount of information. Recently, there has been a proliferation of systematic reviews, which has created the need for an overview of systematic reviews [10]. Such overviews identify and summarize systematic reviews and evaluate their methodological quality. Thus, this overview aims to collate and summarize information for fall interventions from different settings (the community, care facilities and hospitals) in a single publication to serve as a guide for clinicians and other healthcare workers.

This study is part of the ONTOP (Optimal Evidence-Based Non-drug Therapies in Older People) project, a work-package of a European Union funded FP7 research project named SENATOR (Software ENgine for the Assessment & Optimization of drug and non-drug Therapy in Older peRsons) [11]. The aim of the ONTOP project is to provide recommendations, based on the best available evidence from primary trials identified through the systematic reviews, concerning non-pharmacological interventions useful to prevent and/or treat common geriatric conditions [12–18]. In this Overview of Systematic Reviews, we searched 7 electronic databases (the Cochrane Database of Systematic Reviews, PubMed, PsycINFO, EMBASE, CINAHL, PEDRO and TRIP) for systematic reviews and meta-analyses of any non-pharmacological intervention to prevent falls amongst older people, published from 2009 to 2015. This overview summarizes the identified reviews and organizes the interventions according to the ProFaNE (Prevention of Falls Network Europe) taxonomy [19, 20]. The latter includes the various types of measures (exercises, surgery, management of urinary incontinence, fluid or nutrition therapy, psychological, environment/assistive technology, social environment, knowledge and other) performed as single, multiple (fixed combination of interventions given to all subjects) and multifactorial (subjects receive different combinations of interventions based on the evaluation of individual risk factors) interventions. For each ProFaNE intervention category, the reviews are ordered from highest to lowest AMSTAR scores of methodological quality [21] within each intervention context (or ProFaNE Base category).

## Methods

### Search strategy and inclusion criteria for systematic reviews

The search sources included the Cochrane Database of Systematic Reviews, PubMed, PsycINFO, EMBASE, CINAHL, PEDRO and TRIP. We limited our literature search from January 1^st^ 2009 to March 2015. (S1 Fig) Two criteria were considered for further evaluation of an abstract: a) a paper defined as a review or a meta-analysis, b) the mention of any non-pharmacological intervention to prevent falls. (We excluded guidelines.) Vitamin D and calcium supplementation were not considered as non-pharmacological interventions.

Subsequently, full-texts of relevant abstracts were obtained and screened to identify SRs of interest based on:

1. The use of at least one medical literature database;
2. The inclusion of at least one primary randomized controlled trial (RCT);
3. The use of at least one non-pharmacological intervention to prevent falls for people 60+ years of age.

Only studies written in English, Italian or Spanish were considered. We excluded translations of papers from these languages.

We assessed the methodological quality of each systematic review using the AMSTAR (A Measurement Tool to Assess Reviews) instrument that contains 11-items [21]. Two reviewers independently assessed the quality of the SRs and disagreement was resolved by consensus. The kappa statistic between reviewers was 0.94, indicating almost perfect agreement.

This systematic overview has been prepared according to the Preferred reporting items for systematic reviews and meta-analyses (PRISMA) statement (S1 Table) [22].

### Data extraction and management

Extracted data were transferred onto data extraction forms. Information collected included publication year, databases searched, the study population, the non-pharmacological interventions, the number of RCTs included, the outcome measures and the AMSTAR score. Pairs of reviewers independently screened titles, abstracts and full-texts of articles. Disagreement was resolved by discussion. The kappa statistic between reviewers was 0.85, indicating very good agreement.

### Outcome measures

A list of clinically relevant outcomes, for fall prevention, was rated for importance by a panel of international experts to determine which were critical, important and not important [11]. After two rounds of rating, falls, comprising fall rate and number of fallers, was considered a critical outcome. Fall rate was reported as a rate ratio (RaR) where the fall rate of the intervention group was divided by the fall rate of the control group. The fall rate is calculated by dividing the total number of falls by the quantity “person time” during the interval that falls were recorded. Often this rate is expressed as falls per person year. The number of fallers was reported as risk ratios (RR).

## Results

**Fig 1:**
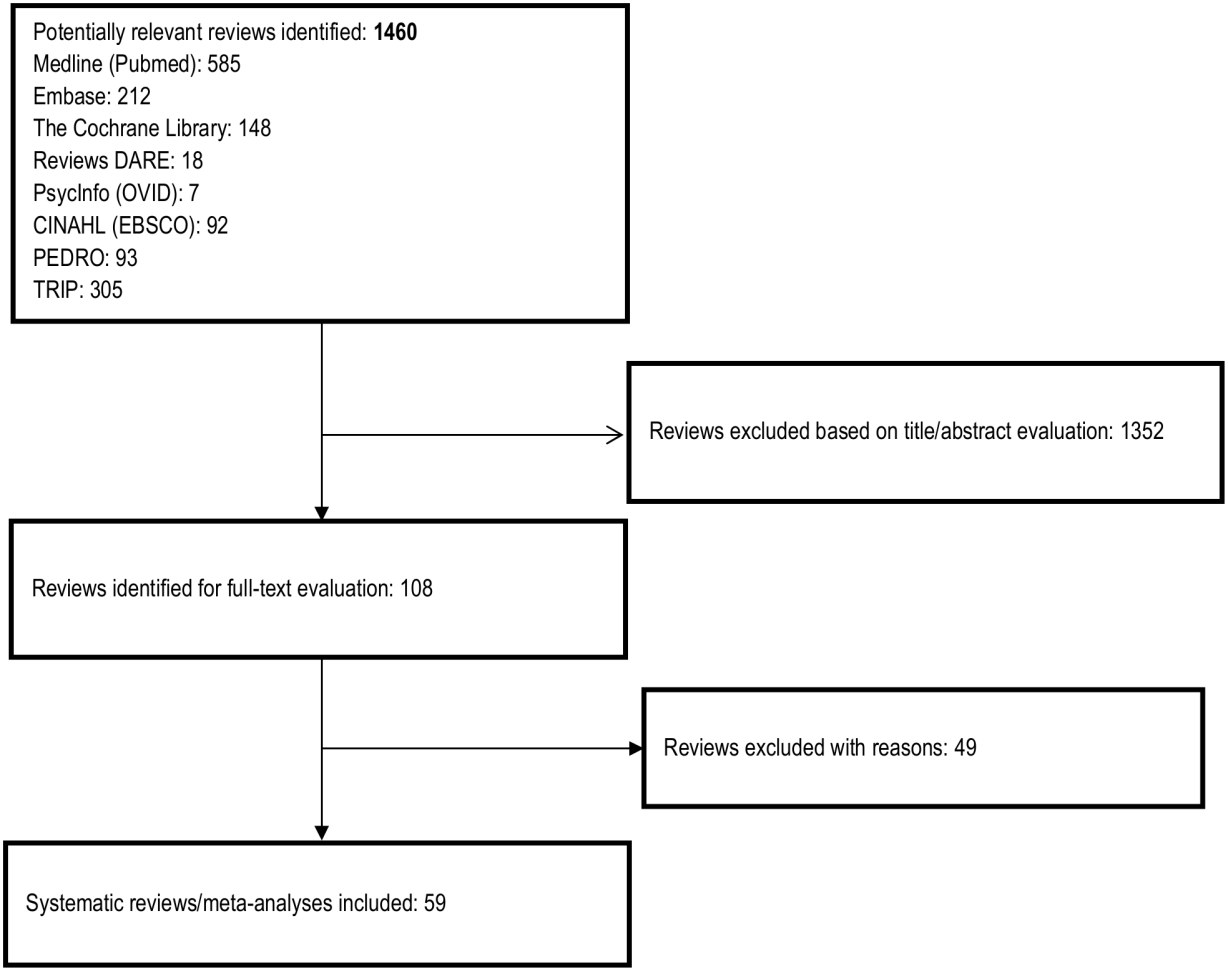
Study screening flow diagram.

Our search identified 1,460 titles of abstracts after excluding duplicates. (Amidst those titles, nine titles were only abstracts[23–31], one full-text could not be found [32] and one record was a Cochrane protocol [33].) Among the 108 potentially relevant publications, 59 were considered eligible for inclusion and 49 were excluded (Fig 1 and S2 Table). Twenty-eight of the included systematic reviews performed meta-analyses [34–59] and thirty-one systematic reviews reported results of the included studies as a narrative summary [60–91]. Fifty-seven out of 59 reviews used Pubmed to search for primary studies. In addition to Pubmed, 45 articles employed CINAHL, 39 papers used the Cochrane Library, 33studies accessed EMBASE, and 17 papers searched PsycINFO.

Eleven SRs were rated as high quality (AMSTAR score 8–11), 37 as medium quality (AMSTAR score 4–7), and 11 as low quality (AMSTAR score 0–3) (S3 Table). A summary of the high and medium quality SRs is presented in order of decreasing AMSTAR score, while the remaining 11 low quality systematic reviews will not be summarized.

The 28 SRs with meta-analyses contained a median of 13 primary RCTs (range 1–159), with an average AMSTAR score of 6.4±2.1, while the 31 SRs without meta-analyses included a median of 10 primary RCTs (range 2–48), with an average AMSTAR score of 4.2±2.3. The difference in mean AMSTAR scores between the two types of reviews was statistically significant (p<0.001; t-test). The average AMSTAR score (±SD) of the reviews was 4.6±2.8 in 2009–2011 and 5.3±2.2 in 2012–2015.

The interventions were classified according to the ProFaNE taxonomy [19, 20] as follows: exercises, surgery, management of urinary incontinence, fluid or nutrition therapy, psychological, environment/assistive technology, social environment, knowledge and other. Exercises encompassed gait, balance, and functional training, strength/resistance training, flexibility, 3D (Tai Chi), general physical activity, endurance or other kinds of exercises not falling within the previous categories. Cataract surgery fell under surgery, as did pacemaker implantation. Environment/assistive technology regarded environmental modifications to increase safety and mobility, low beds, walking aids, hip protectors, identification bracelets, vision assessment/correction, bed alarms and footwear. Staff training was categorized under the social environment. Patient education fell within knowledge. Finally, the interventions organized under “other”, for example, were physical therapy and treatment of postural hypotension. The interventions could be delivered as either single, multiple (fixed combination of interventions given to all subjects) or multifactorial (subjects received different combinations of interventions based on evaluation of individual risk factors) treatments.

Exercise was the most frequent intervention included in the reviews (50), with 18 articles containing exercise as the only intervention [37, 44, 47, 48, 50, 53, 65–67, 69, 71, 72, 76, 79, 84, 87, 88, 91] (S4 Table). The next most frequent interventions fell under Environment in the form of environmental modifications (28 articles) [35, 38–40, 45, 46, 51, 52, 54–59, 63, 68, 70, 73, 74, 77, 78, 80–83, 86, 92], assistive and protective aids (24 reviews) [35, 38–40, 42, 43, 45, 51, 55, 56, 58, 59, 62, 63, 68, 70, 74, 77, 78, 82, 83, 86, 93, 94], staff education (15 SRs) [35, 38, 39, 45, 49, 51, 58, 59, 70, 73, 77, 78, 82, 83, 86] and vision assessment/correction (14 articles) [35, 36, 40, 45, 51, 55–59, 80–82, 86]. Knowledge was the third major category of interventions as patient education (23 papers) [35, 38, 39, 43, 45, 46, 49, 52, 54, 56–59, 68, 70, 73, 74, 77, 78, 81, 82, 86, 89].

In systematic reviews that performed meta-analyses, the estimated effects of the interventions are reported as rate ratio (RaR) for falling rate and risk ratio (RR) for number of fallers.

### Exercises

#### Community-dwelling

A number of reviews are based on studies, themselves based on recruitment location (the ProFaNE Taxonomy Base), where being community-dwelling was the primary feature of the population.

**Fig 2:**
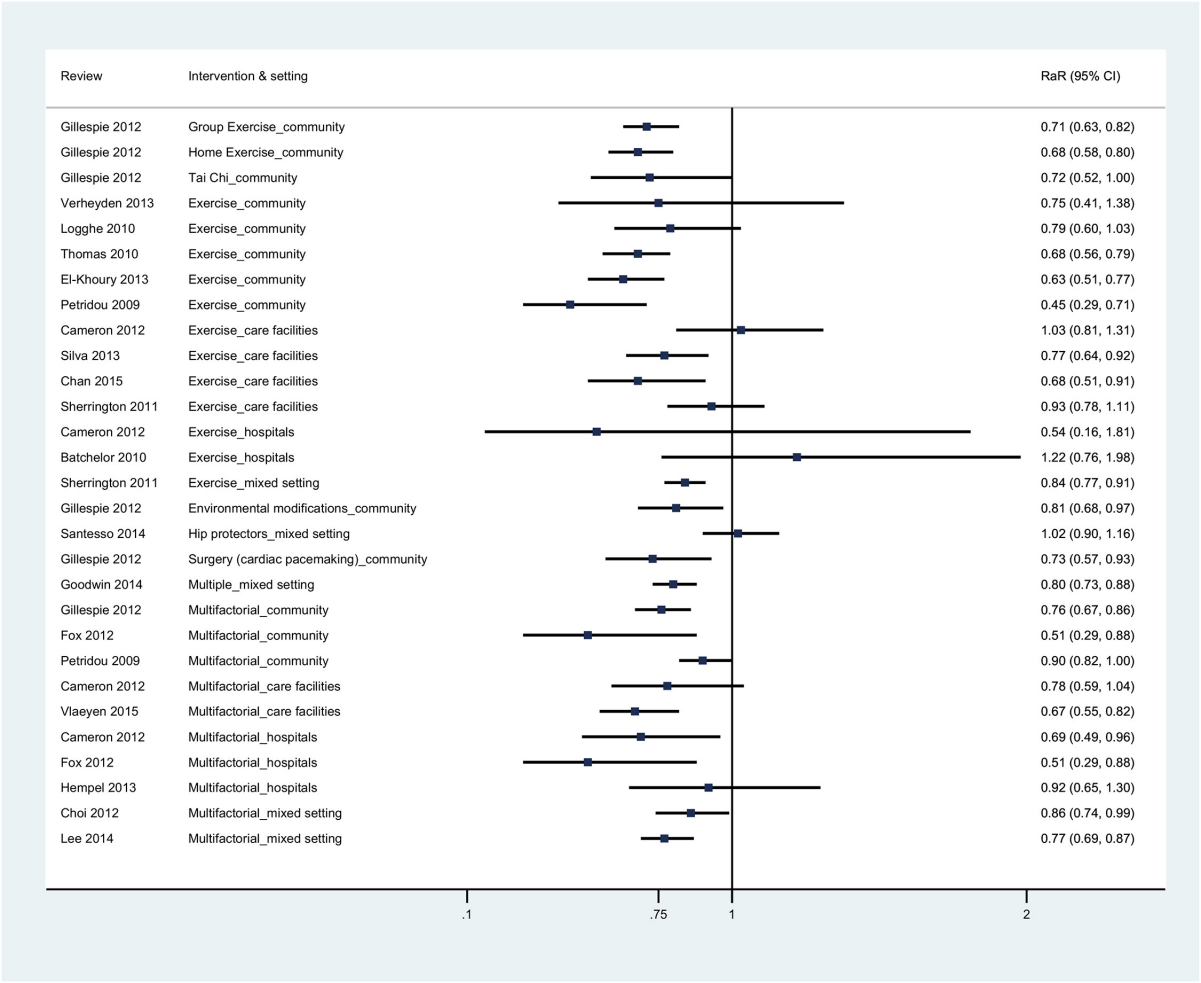
Estimated effects (fall rate) of fall prevention interventions derived from published meta-analyses in different settings. Data are expressed as fall rate ratio (RaR) and 95% confidence interval (95% CI).

**Fig 3:**
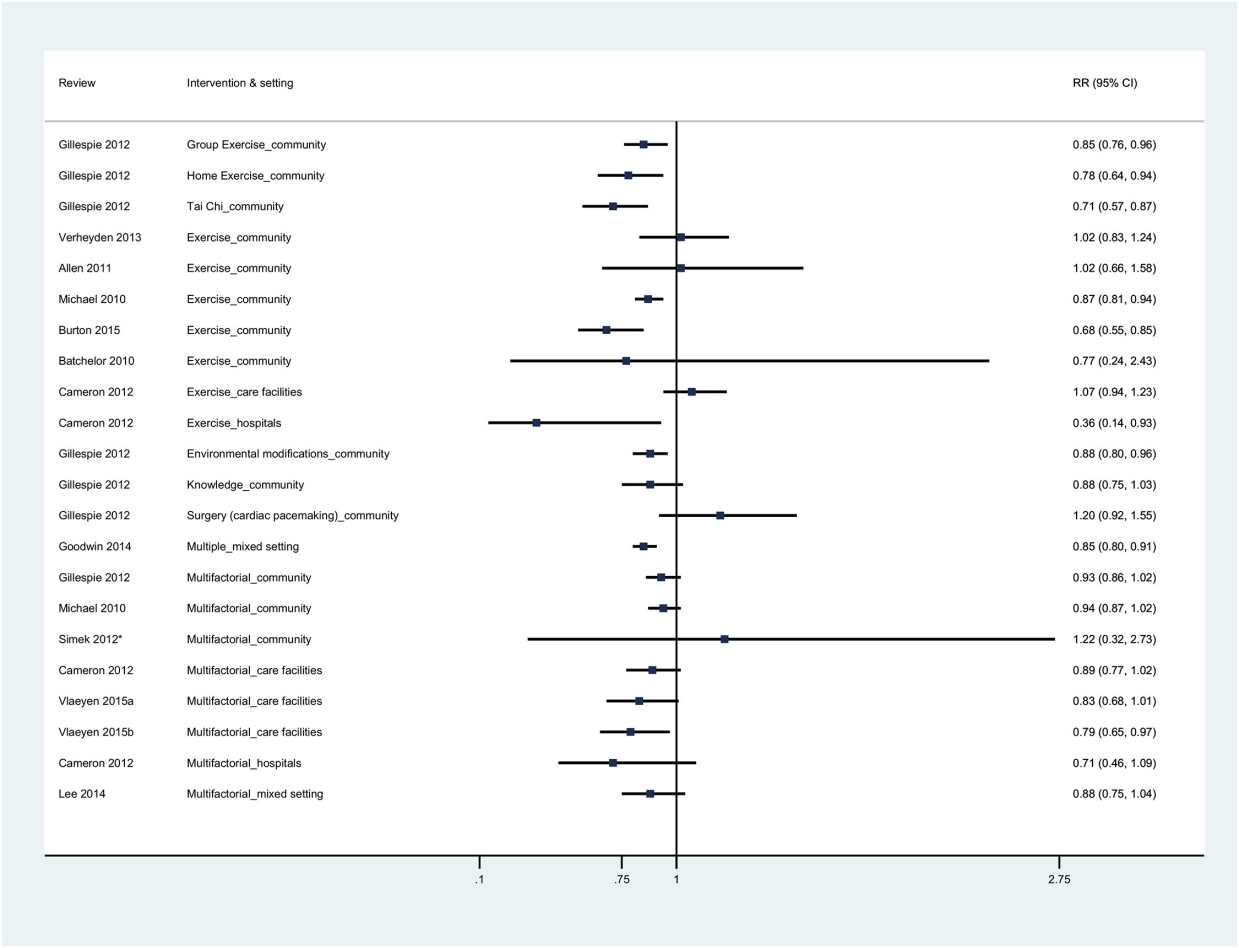
Estimated effects (number of fallers) of fall prevention interventions derived from published meta-analyses in different settings. Data are expressed as risk ratio (RR) and 95% confidence interval (95% CI). *Simek 2012 expressed the estimated effect as an OR (Odds Ratio); Vlaeyen 2015a (number of fallers); Vlaeyen 2015b (number of recurrent fallers).

Gillespie et al 2012 [35] evaluated fall prevention measures, for older people living in the community (AMSTAR = 10). They included 159 RCTs (79,193 participants) that had investigated various types and combinations of interventions. Exercises alone (59 trials) were the most frequent intervention. Falls decreased with multi-element exercise in groups (fall rate, RaR 0.71, 95% CI 0.63–0.82; 16 trials, 3,622 participants; number of fallers, RR 0.85, 95% CI 0.76–0.96; 22 trials, 5,333 participants) (Figs 2 and 3). Levels of heterogeneity were moderate (fall rate: I^2^ = 48%, p = 0.02; number of fallers: I^2^ = 50%, P = 0.004). A similar reduction in falls was observed with multi-element exercise carried out individually at home (fall rate RaR 0.68, 95% CI 0.58–0.80; 7 trials, 951 participants) number of fallers RR 0.78, 95% CI 0.64–0.94; 6 trials, 714 participants). In this case, heterogeneity was absent (fall rate: I^2^ = 0%, P = 0.97; number of fallers: I^2^ = 0%, P = 0.86). Results with Tai Chi were mixed. The number of fallers diminished by 29% (RR 0.71, 95% CI 0.57–0.87; 6 trials, 1,625 participants), whereas the decline in fall rate was not statistically significant (RaR 0.72, 95% CI 0.52–1.00;5 trials, 1563 participants). Heterogeneity was noticeable in the case of fall rate (I^2^ = 72%, P = 0.01), but was not statistically significant for the number of fallers (I^2^ = 41%, P = 0.13). In order to explore the heterogeneity, the authors divided the trials by the risk of falling at recruitment. This subgroup analysis revealed a statistically significant decrease in fall rate, and a reduction in the heterogeneity, only when trials, that had not selected participants for high risk of falling, were combined. (Trials with participants not selected for high risk of falling, RaR 0.59, 95% CI 0.45–0.76; I^2^ = 11%, P = 0.33; 3 trials; trials with participants selected for high risk of falling, RaR 0.95, 95% CI 0.62–1.46; I^2^ = 70%, P = 0.07; 2 trials). In the case of number of fallers, only trials, that had not selected participants for higher risk of falling, demonstrated a statistically significant decline in the number of fallers (trials not selected for higher risk of falling, RR 0.58, CI 0.46–0.74; I^2^ = 0%, P = 0.62; 4 trials; trials selected for higher risk of falling, RR 0.85, 95% CI 0.71–1.01; I^2^ = 0%, P = 0.36; 2 trials). Overall, falls decreased with multicomponent exercise, performed in groups or individually at home, and with Tai Chi.

In Logghe et al 2010 [44] (AMSTAR = 6), Tai Chi, in older people living in the community, did not reduce the fall incidence rate ratio (IRR 0.79, 95% CI 0.60–1.03; I^2^ = 68.6%, P not reported; 5 trials) when compared to non-exercise controls, although when compared to exercise controls (stretching and resistance training), falls declined (IRR 0.51, 95% CI 0.38–0.68; I^2^not reported; 2 trials).

Michael et al 2010 [45] (AMSTAR = 6), assessed 54 RCTs utilizing exercises, multifactorial interventions, vitamin D administration, vision correction, medication assessment, home hazard modification and behavioral counseling as fall prevention measures for community-living older adults. In a meta-analysis, exercises were correlated with a decreased number of fallers (RR 0.87, 95% CI 0.81–0.94; I^2^ = 4.2%, P = 0.41; 15 trials, 3,568 participants).

Thomas et al 2010 [48] (AMSTAR = 6), evaluated 5 RCTs and 1 CCT employing the Otago exercise program[95], composed of strength and balance exercises performed at home, for older people living in the community. Exercise was associated with a decline in fall rate (incidence rate ratio 0.68, 95% CI 0.56–0.79; I^2^ = 0%, P = 0.71;).

El-Khoury et al 2013 [47] (AMSTAR = 5), contained 17 RCTs that examined the effect of exercises on fall prevention of older people living in the community. The fall rate for all injurious falls diminished with exercises (RaR 0.63, 95% CI 0.51–0.77; I^2^ = 50%, P = 0.04; 10 trials).

Petridou et al 2009 [52] (AMSTAR = 4), assessed 10 RCTs that examined exercises alone and as part of multifactorial interventions (exercises, environment/assistive technology, medication review and physical therapy) to avoid falls of older adults living in the community. The pooled estimate for exercises alone was associated with a significant decline in falls (RR 0.45, 95% CI 0.29–0.71; Cochran Q test 18.5 (p<0.001); 5 trials).

#### Residential/nursing care and hospitals

A number of reviews are based on studies, themselves based on recruitment location (the ProFaNE Taxonomy Base), where being in residential/nursing care or hospital community was the primary feature of the population.

Cameron et al 2012 [38](AMSTAR = 8), which is an update of Cameron et al 2010 [39], evaluated diverse types and combinations of ProFaNE interventions for fall prevention of older people in care facilities and hospitals. This systematic review assessed 60 trials (60,345 participants) of which 43 studies (30,373 participants) were carried out in care facilities and 17 RCTs (29,972 participants) were performed in hospitals. In care facilities, exercise had no effect on falls (fall rate RaR 1.03, 95% CI 0.81–1.31; I^2^ = 70%, P = 0.002; 8 trials, 1,844 participants; number of fallers RR 1.07, 95% CI 0.94–1.23; I^2^ = 5%, P = 0.39; 8 trials, 1,887 participants). In hospital subacute wards, supplementary physiotherapy, consisting of supervised exercises, did not statistically significantly decrease fall rate (RaR 0.54, 95% CI 0.16–1.81;1 trial, 54 participants), but was associated with a decline in number of fallers (RR 0.36, 95% CI 0.14–0.93; I^2^ = 0%, P = 0.46; 2 trials,83 participants).

Silva et al 2013 [51] (AMSTAR = 5), investigated the effect of exercises on falls amongst older people in long-term care facilities, including 12 RCTs that examined both single exercises (8 trials; 2 only using Tai Chi) and exercises as part of multifactorial interventions (4 trials) comprising environment/assistive technology, social environment, knowledge and medication review. A meta-analysis of exercises alone and as part of multifactorial interventions, revealed that fall RR declined, although with substantial heterogeneity (0.77, 95% CI 0.64–0.92; I^2^ = 72, P< 0.001; 12 trials, 1,292 participants).

Sherrington et al 2011 [53] (AMSTAR = 5) reported that a meta-analysis of 15 RCTs of exercise, as a single intervention, did not affect fall rate in older people living in care facilities (RaR 0.93, 95% CI 0.78–1.11; neither I^2^ nor number of participants were reported).

#### Mixed residential locations

Gschwind et al 2011 [63](AMSTAR = 6) examined RCTs and non-experimental studies employing exercises, home assessment and modification, of older people in various settings (community and care facilities). The authors described 6 individual studies, with the highest PEDRO score [96] and their results. Due to the large number of exercise interventions and the varied situations in which this type of intervention was performed, the authors were unable to reach any conclusions.

Ishigaki et al 2014 [87] (AMSTAR = 6) included 12 RCTs, of which 2 were cluster randomized, that investigated lower limb muscle strength exercises to prevent falls amongst older adults living at home, in the hospital or a care facility. Although this was a narrative review, because the studies could not be combined due to their heterogeneity, the authors concluded that based on the methodologically high quality studies, this type of exercise is effective for fall prevention in older people.

Sherrington et al 2011 [53] (AMSTAR = 5) pooled 54 RCTs of exercise as a single intervention to prevent falls in older adults living at home and in care facilities, and found that fall rate statistically significantly declined (RaR 0.84, 95% CI 0.77–0.91; the number of participants were not reported), although there was substantial heterogeneity (I^2^ = 56%, P = 0.000).

Lam et al 2012 [65] (AMSTAR = 5), assessed 13 RCTs that had investigated the effect of whole-body vibration (WBV), a type of balance exercise, on older subjects, community-dwelling and living in nursing homes. Most studies evaluated balance and mobility, while 1 RCT found that there was a statistically significant decrease in fall frequency when WBV was combined with exercise (data not shown).

Low et al 2009 [66] (AMSTAR = 5), included 7 RCTs that tested Tai Chi to prevent falls in older adults living in the community and in long-term facilities. Due to the heterogeneity among the studies, the authors did not perform a meta-analysis, but reported the results of each trial. They concluded that this form of exercise has shown promise to reduce falls among the older population as long as they are relatively young and non-frail.

Sitjà-Rabert et al 2012 [67] (AMSTAR = 5), evaluated 16 RCTs that employed WBV for older adults living in the community and in nursing homes. The majority of the trials appraised muscle strength, mobility and balance. The data were not reported for the single trial that studied falls and the authors did not reach any conclusions regarding this outcome.

Schoene et al 2014 [89] (AMSTAR = 5) included 37 studies, of which at least 15 were RCTs, that investigated the effect of interactive cognitive motor training (subjects perform exercises while interacting with a computer interface) on falls and fall risk factors of older people living at home, in independent living facilities, in care facilities and in the hospital. This was a narrative review, because the studies could not be combined due to their heterogeneity. One small RCT (70 participants) reported that fewer people fell in the intervention group.

Gregory et al 2009 [72] (AMSTAR = 4), assessed 6 RCTs that investigated Tai Chi to prevent falls in older adults living in the community and in long-term facilities. The results of the trials were individually described. The authors concluded that this type of exercise may be beneficial for non-frail older people.

Leung et al 2011 [50] (AMSTAR = 4), appraised 13 randomized controlled trials that investigated Tai Chi as a fall prevention measure for older adults living in the community and long-term care centers. Falls (5 trials) and balance were measured. The authors reported that 4 trials found a reduction in falls, and 1 study found no difference in falls, for healthy older adults. One of the trials also reported contrasting results for frail older subjects.

#### Disease-specific populations

A number of reviews are based on studies in which recruitment was based on specific disease or impairment criteria, rather than being based on residential status.

Verheyden et al 2013 [36] (AMSTAR = 9) assessed fall prevention measures (exercises, vitamin D administration and single-lens distance eyeglasses), following stroke (acute, subacute and chronic phases) in 10 RCTs, comprising 1,004 participants. Exercise had no effect, during any stage subsequent to stroke, on fall rate (acute and subacute phase: RaR 0.92, 95% CI 0.45–1.90; 1 trial, 95 participants; chronic stage: RaR 0.75, 95% CI 0.41–1.38; I^2^ = 72%, P = 0.01; 4 trials,412 participants) and number of fallers (acute and subacute phase: RR 1.19, 95% CI 0.83–1.71; 1 trial, 95 participants; chronic stage: RR 1.02, 95% CI 0.83–1.24; I^2^ = 15%, P = 0.32; 6 trials, 616 participants).

Batchelor et al 2010 [46] (AMSTAR = 5), included 13 RCTs that investigated exercises, environment/assistive technology, social environment and medication review for older people following stroke. Exercise had no effect on falls (fall rate RaR 1.22, 95% CI, 0.76–1.98; I^2^ not reported; 2 RCTs, 119 participants; number of fallers RR 0.77, 95% CI, 0.24–2.43; I^2^ not reported; 2 RCTs, 224 participants).

Allen et al 2011 [37] considered studies that evaluated exercise therapy in subjects with Parkinson’s disease. The review had an AMSTAR score of 8 and included 2 RCTs (250 participants). In a meta-analysis of the RCTs, exercises had no effect on the number of fallers (RR 1.02, 95% CI 0.66–1.58). (The level of heterogeneity was moderate, but non-significant (I^2^ = 49%, P = 0.16).)

Chan et al 2015 [91] (AMSTAR = 6) examined the effect of exercises, as a single intervention, to prevent falls of cognitively impaired older adults in care facilities and living at home. These authors combined data from 4 trials involving subjects with cognitive problems and subgroup data of cognitively impaired older adults from 3 trials. This type of intervention was associated with a decline in fall rate (RaR 0.68, 95% CI 0.51–0.91; 7 RCTs, 688 participants), although the heterogeneity was substantial (I^2^ = 79%, P<0.001).

Burton et al 2015[55] (AMSTAR = 6) included 3 RCTs of exercises to prevent falls of community-dwelling older people with dementia. Exercises were part of multifactorial interventions (1 RCT) or as a single intervention, in a group or individually. Two meta-analyses of 2 RCTs (271 participants) of exercise alone, revealed a reduction in the number of falls (Mean difference -1.06, 95% CI -1.67 –-0.46; I^2^ = 0%, P = 0.95) and of fallers (RR 0.68, 95% CI 0.55–0.85; I^2^ = 0%, P = 0.99).

Winter et al 2013 [74] (AMSTAR = 5), evaluated 5 RCTs that studied single and multifactorial interventions composed of exercises, knowledge, environment/assistive technology, psychological interventions and medication review to prevent falls of older people, with cognitive impairment, living in the community. One trial compared 3 single component interventions (exercise training, education and home safety assessment and modification), but there was no statistically significant difference in fall rate among the 3 intervention groups.

Gleeson et al 2014[90] (AMSTAR = 6) included 4 RCTs consisting of 3 trials of exercise as a single intervention in residential facilities and 1 study of exercise alone or together with home safety assessment/modification at home, of older adults with untreatable visual impairment. The 2 RCTs that measured falls could not be combined due to the heterogeneity of interventions and settings. Exercise had no effect on falls in either study, but there was a non-statistically significant reduction of falls in the residential facility study.

Cadore et al 2013 [69] (AMSTAR = 4), consisted of 20 randomized controlled trials that investigated different types of exercise to prevent falls in frail older people. The outcomes measured were falls, balance, mobility, muscle strength and oxygen consumption (VO_2 max_). Falls were evaluated in 10 of the trials. The authors reported that falls declined in 7 of the studies (an average decrease of 22–58%), while no effect was demonstrated in 3 trials.

de Kam et al 2009 [71] (AMSTAR = 4), identified 28 randomized controlled trials that used exercises to decrease fractures due to falls, and their associated risk factors, in subjects with low bone density. The outcomes measured comprised falls, fall-associated fractures, bone-mineral density, muscle strength, balance and walking velocity. The intervention reduced falls in 3 RCTs in which this outcome was measured.

Voigt-Radloff et al 2013 [68] (AMSTAR = 5), included 48 RCTs that used exercises, environmental modification and multifactorial interventions as fall prevention measures for older people after stroke, in semi-acute wards, rehabilitation units, residential care and the community, hip-fracture patients in the hospital and in rehabilitation wards, residents of psychogeriatric wards, community-dwelling older adults, with and without dementia, Parkinson’s patients and older adults in long-term care homes. In 1 trial of exercises as a single intervention, falls of community-living older people, with ≥1 fall risk factor, were unchanged.

In summary, in community-dwelling adults, exercise had a consistent effect in reducing both fall rate and the number of fallers (5 out of 6 reviews, including Gillespie 2012, which had a high methodological rating (AMSTAR score = 10) and contained the largest number of RCTs and participants [35]). Exercise was reported to be ineffective in care facilities in two reviews (Sherrington 2011 [53] and Cameron 2012 [38]) and effective in another (Silva 2013 [51]), but the latter combined trials of exercise as a single intervention, with trials of exercise as part of a multifactorial intervention, making it difficult to draw conclusions. In hospital subacute wards, physical exercise decreased the number of fallers, but not the fall rate (1 review). Regarding disease-specific patient populations, exercise did not affect falls in people who had experienced a stroke (3 reviews) and in subjects with Parkinson’s disease, whereas 2 out of 3 reviews reported that falls were reduced in cognitively-impaired older adults, as well as in older frail (1 review) and osteoporotic subjects (1 review).

### Environment/assistive technology

#### Environmental modifications: community-dwelling

Gillespie et al 2012 [35] (AMSTAR = 10), reported that assessment and modification of home safety was effective in reducing falls (fall rate (RaR 0.81, 95% CI 0.68–0.97; I^2^ = 64%, p = 0.02; 6 trials, 4,208 participants) and the number of fallers (RR 0.88, 95% CI 0.80–0.96; I^2^ = 0%, p = 0.73; 7 trials, 4,051 participants)). The authors performed two separate subgroup analyses to determine the influence of the participants’ risk of falling (high versus low) and the healthcare professional who delivered the intervention (occupational therapist versus non-occupational therapist). The results of the subgroup analyses revealed that this type of intervention was effective in reducing fall rate and number of fallers only in individuals with a greater risk of falling or only when administered by an occupational therapist.

Gschwind et al 2011 [63] (AMSTAR = 6) reported that only 2 out of 6 randomized controlled trials that investigated home assessment and modification of older people living in the community and care facilities reported a reduction in falls.

Michael et al 2010 [45] (AMSTAR = 6) reported on three RCTs that examined home hazard modification, 2 of which were combined with behavioral counseling, for community-living older people. Only 1 of the RCTs combined with psychological support demonstrated a statistically significant reduction in the number of fallers.

The 1 year fall rate, of older people living at home, decreased following environmental modification by trained occupational therapists (results from 1 RCT described; Voigt-Radloff et al 2013 [68] (AMSTAR = 5)).

#### Environmental modifications: residential/nursing care and hospitals

Anderson et al 2012 [61] (AMSTAR = 9), assessed 2 RCTs that investigated the effect of bed modifications (low height beds and bed exit alarms) on patient falls, fall-related injuries and bed-related injuries in hospitals (acute and subacute wards) and care facilities. Due to the diversity of the interventions, the 2 studies could not be combined and the results were described for each. The investigators thought that the effectiveness of the measures to avert bed-associated patient injuries were inconclusive.

Cameron et al 2012 [38] (AMSTAR = 8), reported 5 randomized controlled trials that investigated various environment/assistive technologies (a wireless position-monitoring device, carpeted floors, low beds, blue identification bracelets and a bed exit alarm) conducted to avoid falls of older people in care facilities and hospitals (acute and subacute wards) were not pooled. None of the studies reduced falls and 1 trial found that floor carpeting in a subacute hospital ward led to increased fall rate.

One RCT that investigated environmental modification, in hospitals, did not affect fall rate or number of fallers of older people following stroke (Batchelor et al 2010 [46] (AMSTAR = 5)).

Choi et al 2011 [70] (AMSTAR = 4), included 9 RCTs that employed single and multifactorial interventions to reduce falls of older people in hospital, sub-acute wards, rehabilitation wards and long-stay geriatric care units. In the 1 trial that examined environment modification, falls were lower on vinyl floors than on floors with carpeting.

Kosse et al 2013 [73] (AMSTAR = 4), examined 3 RCTs that made use of sensor technologies alone or as part of a multifactorial intervention, comprising exercises, knowledge, environmental modification, social environment and medicine review, for fall prevention of older people in hospitals (acute care, elderly care and geriatric stroke rehabilitation wards). There were no significant differences in number of falls in 2 RCTs that investigated bed and chair sensors. (The trial results were described individually).

#### Hip protectors: mixed locations

Santesso et al 2014 (AMSTAR = 10) [94] evaluated hip protectors for older people living in nursing homes or at home in which fall rate and fractures (hip, pelvic and other types) were measured. (Santesso et al 2014 is an update of Gillespie et al 2010 [34]). The authors included 19 randomized controlled trials, 10 of which used cluster randomization of the care structures. A meta-analysis revealed that this type of intervention did not influence falls (fall rate RaR 1.02, 95% CI 0.90–1.16; I^2^ = 92%, p<0.00001; 16 trials (13 nursing home RCTs, 3 community RCTs)).

#### Hip protectors: residential/nursing care and hospitals

Combes et al 2013 [62] (AMSTAR = 5), assessed hip protectors for older people in residential care facilities (4 RCTs), similar to the previous systematic review. Each study was described without analyzing pooled data. The authors recommended the use of hip protectors, even though the evidence was not conclusive.

#### Vision improvement: community-dwelling

Gillespie et al 2012 [35] (AMSTAR = 10), evaluated the effect of vision improvement to avoid falling in older people living at home. Due to the variability of the interventions, the results of the 3 RCTs were not pooled, but were described separately. Overall, this class of intervention did not influence falls (falling rate or number of fallers), although in 1 of the trials, falls rose (fall rate RaR 1.57, 95% CI 1.19–2.06 and number of fallers RR 1.54, 95% CI 1.24–1.91). Older people appear to be at increased risk during the period following changes to their prescription and while adjusting to new glasses and/or multifocal glasses.

Neither fall rate nor number of fallers decreased by substituting single lens distance eyeglasses for multifocal glasses following stroke of older community-dwellers (1 RCT; Verheyden et al 2013 [36] (AMSTAR = 9)).

Two RCTs of vision correction did not influence number of fallers of community-living older people (Michael et al 2010 [45] (AMSTAR = 6)). The studies were summarized without performing a meta-analysis.

#### Social environment: residential/nursing care and hospitals

Overall, neither staff training (4 RCTs not pooled) nor service model change (6 RCTs not combined) in care facilities and hospitals reduced fall rate or the number of fallers, although the investigators of 2 trials reported fewer falls associated with service model change in care facilities (Cameron et al 2012 [35] (AMSTAR = 10)).

#### Social environment: mixed locations

Four RCTs, using the same population, examined home versus standard rehabilitation, but did not demonstrate an effect on fall rate or number of fallers of older people following stroke (Batchelor et al 2010 [46] (AMSTAR = 5)).

#### Footwear modification: community-dwelling

Gillespie et al 2012 [35] (AMSTAR = 10), assessed 2 RCTs, not combined, that modified footwear as a fall prevention measure, for community-dwelling older adults. A non-slip shoe device diminished outside fall rate in winter, while insoles to improve balance had no effect on the number of fallers.

#### Identification bracelets: residential/nursing care and hospitals

Using identification bracelets had no effect on fall reduction of high risk patients in a rehabilitation hospital (1 RCT described; Choi et al 2011 [70] (AMSTAR = 4)).

To summarize, 4 SRs reported that environmental modification reduced falls, in community-dwelling older adults, and in addition, the largest review with a high methodological rating (AMSTAR score = 10) (Gillespie 2012 [35]) found that this type of intervention was effective only in older subjects with a greater risk of falling or when delivered by an occupational therapist. In care facilities and hospitals, falls did not change (4 out of 5 reviews), except when vinyl floors were used in place of carpeting (1 review). Hip protectors had no effect on falls in any setting (2 reviews). Vision correction did not alter falls in community-dwelling older people (3 SRs). Social environment modifications (staff training and service model changes) did not influence falls in care facilities and hospitals. In winter, falls outside declined with a non-slip shoe device in community-dwelling older adults (1 review). Finally, identification bracelets were unable to influence falls in care facilities and hospitals (1 review).

### Knowledge or educational interventions

#### Community-dwelling

Increased knowledge about fall prevention had no effect on fall rate (1 trial) or number of fallers (RR 0.88, 95% CI 0.75–1.03; I^2^ = 0%, P = 0.61; 4 RCTs, 2,555 participants) of older adults living at home (Gillespie et al 2012 [35], (AMSTAR = 10)).

#### Residential/nursing care and hospitals

Two randomized controlled trials, which could not be combined, investigated patient education as a means to prevent falls of older people in care facilities and hospitals (Cameron et al 2012 [38] (AMSTAR = 8)). One of the trials targeting individual fall risk factors, of high falling risk patients, was associated with a decrease in fall rate, while the second study had no effect on either fall rate or number of fallers.

### Surgery

#### Community-dwelling

Gillespie et al 2012 [35](AMSTAR = 10), evaluated 2 types of surgery, cardiac pacing and cataract surgery, to prevent falls of older adults living in the community. In individuals with cardio-inhibitory carotid sinus hypersensitivity, the use of cardiac pacemakers was associated with reduced fall rate (RaR 0.73, 95% CI 0.57–0.93; I^2^ = 51%, p = 0.13; 3 trials, 349 participants), but not the number of fallers (RR 1.20,95% CI 0.92–1.55; I^2^ = 0%, p = 0.58;2 trials, 178 participants). Regarding cataract surgery, removal of the cataract in the first eye in women, but not in the second eye, was correlated with decreased fall rate (RaR 0.66, 95% CI 0.45–0.95; 1 RCT, 306 participants).

Desapriya et al 2010 [41] (AMSTAR = 7), identified 3 RCTs that employed expedited cataract surgery. A meta-analysis of the 2 trials that reported falls data, did not reveal a statistically significant reduction of fall rate compared to routine cataract surgery (OR 0.81, 95% CI 0.55–1.17; I^2^ = 0%, p = 0.35).

Ishikawa et al 2013 [64] (AMSTAR = 7), evaluated 3 RCTs that investigated second-eye cataract surgery in older people, but only 1 trial examined falls. The authors concluded that the evidence was limited.

Michael et al 2010 [45] (AMSTAR = 6), assessed 2 RCTs of cataract surgery as fall prevention measures for community-living older people. The number of fallers did not decrease with this intervention.

There is evidence that cardiac pacing in subjects with cardio-inhibitory carotid sinus hypersensitivity and cataract surgery can reduce falls, although the evidence for the latter surgical intervention was not consistent.

### Psychological interventions

#### Community-dwelling

The cognitive behavioral interventions tested in 2 RCTs, had no effect on either fall rate or number of fallers of older people living at home (Gillespie et al 2012 [35] (AMSTAR = 10)).

One RCT that employed behavioral counseling for community-living older people was summarized, without reporting the authors’ conclusions, in Michael et al 2010 [45] (AMSTAR = 6).

An RCT that investigated a cognitive behavioral group intervention, of 9 weekly sessions, found a significant reduction in recurrent falling of community-living older adults with cognitive impairment (Winter et al 2013 [74] (AMSTAR = 5)).

### Management of urinary incontinence

#### Residential/nursing care and hospitals

Batchelor et al 2013[85] (AMSTAR = 6) included 2 RCTs of urinary incontinence management strategies (non-pharmacological and pharmacological treatments) to prevent falls of older adults in care facilities. This was a narrative review, because the studies could not be combined due to their heterogeneity. One RCT reported that fewer people fell following a multiple non-pharmacological intervention comprising management of urinary incontinence, fluid therapy and exercise.

### Other

#### Community-dwelling

In 1 RCT, there was an increase in falls of older adults living at home, who had fallen ≥1, following 7 weeks of occupational group therapy, one additional session and one home visit (Voigt-Radloff et al 2013 [68] (AMSTAR = 5)).

Two RCTs that used multifaceted assessment of falls risk of older adults, with cognitive impairment, living at home, found discordant effects on falls (Winter et al 2013 [74] (AMSTAR = 5)).

#### Residential/nursing care and hospitals

Neither lavender patches (1 trial, 145 subjects) nor increased exposure to sunlight (1 trial, 395 participants) affected fall rate or number of fallers of older subjects in care facilities (Cameron et al 2012 [38] (AMSTAR = 8)).

Neyens et al 2011 [78] (AMSTAR = 4), included 20 RCTs that evaluated single and multifactorial interventions (exercises, management of urinary incontinence, hip protectors, patient and staff education, physical therapy, medication review, vitamin D supplementation) to avert falls of older adults in long-term care facilities. A single RCT that examined individual physical therapy to reduce falls was described.

### Multiple interventions

This category of intervention includes all those studies in which the different interventions are administered to all participants without assessing individual risk factors.

#### Community-dwelling

In Gillespie et al 2012 (AMSTAR = 10) [35], 18 trials investigated different types of multiple interventions, each consisting of different combinations of specific interventions, such as exercise, home safety, vision assessment, education, clinical assessment, multifactorial assessment, nutrition, nutritional supplement, cognitive behavioral therapy, podiatry and rehabilitation. The most frequent component of the multiple interventions was exercise (n = 14 trials). Eight out of 19 combinations of multiple interventions were able to reduce fall rate and 5 out of 18 combinations of multiple interventions led to fewer older people falling at home. Gillespie et al 2012 analyzed combinations of interventions separately, since no two multiple interventions were the same, but few multiple interventions were effective.

#### Mixed locations

Goodwin et al 2014[56] (AMSTAR = 8) included 17 RCTs (parallel group, cluster and factorial randomization) of multiple interventions (exercises together with vitamin D, calcium, management of urinary incontinence, fluid or nutritional therapy, psychological measures, environment/assistive aids, knowledge, vision improvement and others; vitamin D with calcium and nutritional supplements or calcium with exposure to sunlight) tested in 22 intervention groups to prevent falls of older people in different settings (community (n = 10 trials), outpatient clinics (n = 2 trials), hospital (n = 1 trial), care facilities (n = 2 trials), or not specified (n = 2 trials)). Nineteen of the twenty-two intervention groups employed exercise. By pooling the RCTs in a meta-analysis, the authors observed a decrease in fall rate ratio (RaR 0.80, 95% CI 0.73–0.88; I^2^ = 19%, P = 0.23; 11 trials) and the number of people falling (RR 0.85, 95% CI 0.80–0.91; I^2^ = 0%, P = 0.80; 12 trials).

#### Residential/nursing care and hospitals

Neither supervised exercises combined with fluids and regular toileting (1 RCT, 190 participants), nor increased sunlight paired with calcium supplementation (1 trial, 602 residents) influenced fall rate or the number of fallers in older adults in care facilities (Cameron et al 2012 [38] (AMSTAR = 8)).

Neyens et al 2011 [78] (AMSTAR = 4), assessed 5 RCTs of multiple interventions in long-term care facilities (1 study of exercise plus incontinence care, 3 RCTs of hip protectors combined with staff and patient education and 1 trial of various interventions (exercise/physical therapy, staff and patient education, environmental/personal safety, protector)). Only the findings of individual trials were reported, without any specific conclusions.

### Multifactorial interventions

In this category of intervention, subjects are assessed for fall risk factors and receive tailored combinations of interventions based on those risk factors.

#### Community-dwelling

Gillespie et al 2012 [35], (AMSTAR = 10) found multifactorial interventions were associated with a decline in fall rate (RaR 0.76, 95% CI 0.67–0.86; I² = 85%, p < 0.00001; 19 trials, 9503 participants), but not the number of fallers (RR 0.93, 95% CI 0.86–1.02;I² = 69%, p < 0.00001; 34 trials, 13,617 participants) in community living older adults.

Michael et al 2010 [45] (AMSTAR = 6) reported that the number of fallers, in community-living older people at high risk of falling, was not reduced by multifactorial interventions (RR 0.94, 95% CI 0.87–1.02; I^2^ = 61.5%, P<0.001; 19 RCTs, 6322 participants).

A multifactorial intervention in 1 RCT, targeting all identified risk factors, had no effect on falls of older adults with cognitive impairment living at home (Winter et al 2013[74] (AMSTAR = 5)).

Simek et al 2012 [54] (AMSTAR = 4), identified 23 randomized controlled trials that investigated adherence to, and effectiveness of, multifactorial intervention (exercise, education, social support, hazard assessment, footwear, and vitamin D supplementation) and home exercise programs, to prevent falls in older people. Combined results, from trials of multifactorial interventions (7 RCTs) and exercise alone (5 RCTs), did not demonstrate an effect on the number of fallers (OR 1.22, 95% CI 0.32–2.73; I^2^ not reported).

Multifactorial interventions were unable to reduce the incidence of falls of older adults living in the community (RR 0.90, 95% CI 0.82–1.00; Cochran Q test 6.9, p = 0.14; 5 RCTs; Petridou et al 2009 [52] (AMSTAR = 4).

#### Residential/nursing care and hospitals

Cameron et al 2012 [38] (AMSTAR = 8) reported that falls (fall rate (7 RCTs, 2,876 participants) and the number of fallers (7 trials, 2,632 subjects)), of older subjects in care facilities, were unaffected by multifactorial interventions. In contrast, this approach was associated with lower fall rate (RaR 0.69, 95% CI 0.49–0.96; I² = 59%, p = 0.06; 4 RCTs, 6,478 participants), and the number of fallers (RR 0.71, 95% CI 0.46–1.09; I² = 43%, p = 0.17; 3 trials, 4,824 subjects), although the latter was not statistically significant, of older patients in hospitals (acute and subacute wards).

Vlaeyen et al 2015[59] (AMSTAR = 8) performed meta-analyses of 13 RCTs (11 trials were clustered randomized) of fall prevention programs in nursing homes, examining the number of falls, fallers and recurrent fallers after single (exercises, staff training, education, vitamin D administration and medication review), multiple (exercises together with management of urinary incontinence and fluid or nutrition therapy) and multifactorial interventions (exercises, medication review, advice to correct orthostatic hypotension, education and environment (evaluation and modification, staff training, hip protectors, vision, footwear)). The authors carried out this investigation, because other meta-analyses, such as Cameron et al 2012 [38], did not detect a decline in the number of falls and fallers. Vlaeyen et al 2015 thought the latter was due to combining trials that investigated people needing different levels of assistance (high and intermediate), as well as using vague definitions of the care contexts (e.g., long-term care facilities, residential care facilities, nursing homes) to identify studies. Using a specific definition of a nursing home, as part of their inclusion criteria to select trials, Vlaeyen et al 2015 reported a statistically significant decrease in the number of falls (RR 0.67, 95% CI 0.55–0.82; I² = 16.7%, P = 0.31; 4 trials) and recurrent fallers (RR 0.79, 95% CI 0.65–0.97; I² = 6.3%, P = 0.36; 4 trials), but a non-statistically significant reduction in fallers (RR 0.83, 95% CI 0.68–1.01; I² = 20%, P = 0.29; 4 trials), only when multifactorial intervention trials were combined in a meta-analysis.

Fox et al 2012 [42] (AMSTAR = 7), assessed 9 randomized controlled trials (6,839 participants) performed in patients admitted to acute geriatric care units. This is a type of multifactorial intervention composed of care activities to avoid deterioration of ADLs, mobility, continence, nutrition, skin integrity, mood, sleep, and cognition, repeated medical review, early rehabilitation, early discharge planning and environmental modifications [97]. Pooled results from 2 trials demonstrated a decline in falls (RR 0.51, 95% CI 0.29–0.88; I^2^ = 0%, P = 0.55; 894 participants).

DiBardino et al 2012 [43] (AMSTAR = 6), investigated the effect of multifactorial interventions (exercise, mobility aid, medication modification, education, bed interventions (e.g. bed alarm, rail adjustment and toileting schedule) as a fall prevention strategy for acute care inpatients. A meta-analysis of 1 RCT, 1 controlled clinical trial (CCT) and 4 pre-post studies revealed that fall rate declined, although at the limit of statistical significance (OR 0.90, 95% CI 0.83–0.99; I^2^ = 0%, P not reported).

Choi et al 2011[70] (AMSTAR = 4), included 9 RCTs that employed multifactorial interventions (exercises, management of urinary incontinence, nutrition therapy, environment/assistive technology, social environment, knowledge and medication review) to reduce falls of older people in the hospital, sub-acute wards, rehabilitation wards and long-stay geriatric care units. The authors only described the results of the primary studies.

Hempel et al 2013 [49] (AMSTAR = 4), contained 59 studies (4 RCTs, of which 2 were cluster randomized, 7 controlled clinical trials, 41 before-after and 7 time series studies with historical controls) that made use of various multifactorial interventions, comprising low beds, bed alarms, non-skid socks and slippers, hip protectors, suitable lighting, side rails, non-skid shower mats, fall prevention poster, patient and family education and restraints to prevent falls of older patients in the hospital (acute, rehabilitation wards). These interventions were not associated with a decrease of the fall incidence rate (IRR 0.92, 95% CI 0.65–1.30; I^2^ = 68%, p not reported; 5 trials).

There was no difference in fall rate, in elderly care wards in hospitals, with a multifactorial intervention (1 RCT, Kosse et al 2013 [73] (AMSTAR = 4)).

Neyens et al 2011 [78] (AMSTAR = 4), included 8 RCTs that evaluated multifactorial and multiple (1 RCT) interventions (exercise/physical therapy, staff and patient education, environmental/personal safety, referral to relevant disciplines, hip protector, supplying/repairing aids, change in medication, vitamin D supplementation) to avert falls of older adults in long-term care facilities. The results and characteristics of individual studies were described qualitatively without performing a meta-analysis.

#### Mixed locations

Choi et al 2012[40] (AMSTAR = 7), studied single (exercises and vitamin D supplementation) and multifactorial (exercises, home and community environmental safety improvement, vision correction, medication review, footwear modification and assistive and protective aids) measures to avoid falls of older adults living at home (14 trials) or in nursing homes (3 RCTs). A meta-analysis combining all 17 RCTs, 2 of which examined single interventions, indicated that fall rate diminished, although at the limit of statistical significance, and with a very high inter-study heterogeneity (RaR 0.86, 95% CI 0.74–0.99; I^2^ = 92%, P not reported).

Lee et al 2014 [58] (AMSTAR = 6) included 13 RCTs, of which 3 were cluster RCTs, that investigated the effect of multifactorial interventions, containing patient education, during hospitalization or post-discharge, on fall rate, number of fallers, injurious falls, number of fallers with injury, rate of hospital readmissions and emergency department visits of older adults. There was a statistically significant decrease in fall rate when different types of studies were combined (RaR 0.77, 95% CI 0.69–0.87; I^2^ = 35.5%, P = 0.092; 14 studies, of which 12 were multifactorial studies (cluster RCT n = 3, RCT n = 8, quasi experimental n = 1, pre-post study n = 2)), while there was a non-statistically significant reduction in the proportion of fallers again when pooling various types of studies (RR 0.88, 95% CI 0.75–1.04; I^2^ = 52.3%, P = 0.014; 13 studies, of which 12 were multifactorial studies (clustered RCT n = 2, RCT n = 7, CCT n = 1, quasi experimental n = 1, pre-post study n = 1, retrospective cohort study n = 1)).

Bunn et al 2014 [86] (AMSTAR = 6) included 17 RCTs, including 8 cluster randomized trials, that investigated single (exercises, social environment, knowledge, multisensory stimulation, lavender olfactory stimulation) or multifactorial (exercises, medication review, management of urinary incontinence, fluid or nutrition therapy, environment/assistive technology, social environment, knowledge) interventions to prevent falls of older people with dementia, cognitive impairment or depression, living at home, in care facilities and in the hospital. This was a narrative review, because the studies could not be combined due to their heterogeneity. Seven trials found a statistically significant reduction in the number of falls/fall rate and 4 studies reported a decrease in the number of fallers.

Voigt-Radloff et al 2013 [68] (AMSTAR = 5), included 6 RCTs that examined multifactorial interventions of older adults in rehabilitation hospitals, nursing homes and living at home. There was no consistent effect on falls among the trials, probably due to the variation in elements comprising the interventions.

In community-dwelling older adults, multifactorial interventions decreased fall rate, but not the number of fallers. In acute and rehabilitation wards of hospitals, multifactorial interventions were reported to reduce falls in 3 SRs, while 2 reviews reported negative results, although the review with a high methodological rating (AMSTAR score = 8) and the largest number of participants (Cameron et al 2012 [38]), reported a reduction in falls. There were contrasting results in care facilities. While Cameron et al 2012 [38] did not find a statistically significant reduction in falls, using a precise definition of a nursing home as part of their inclusion criteria, Vlaeyen et al 2015 [59] reported a reduction in falls.

## Discussion

### Summary of the main results

This overview of systematic reviews has brought together 59 systematic reviews that evaluated non-pharmacological interventions to prevent falls in older people. Eligible articles were identified from comprehensive searches of published reviews using several relevant electronic databases. Rigorous procedures were used for review identification, quality assessment and data extraction.

This overview serves as a guide to the effectiveness of any non-pharmacological intervention consisting of single, multiple and multifactorial measures and their use in different settings including community-dwelling, care facilities (e.g., long-term, residential, nursing homes) and hospitals (acute and sub-acute wards).

The interventions were classified according to the ProFaNE taxonomy, which characterizes existing fall prevention interventions, as well as setting. Reviews are presented from the highest to the lowest AMSTAR score, with a focus on the reviews with the highest quality within each context.

The vast majority of the reviews (n = 50) included studies employing exercise either as a single intervention (n = 18) or combined with other types of interventions. The next most common intervention reviewed was the environment; in terms of environmental modifications (n = 28), assistive and protective aids (n = 24). Knowledge or education was the third major type of intervention, specifically patient education (n = 23) and staff education (n = 15).

### Exercise

Exercise was by far the most effective intervention to reduce falls in older adults, although not all types of exercise were equally effective in all participant groups and in all settings. Effective exercise programs to prevent falls included a combination of demanding and progressive balance exercises carried out in weight-bearing positions, with lower limb strength training (see the description of the Otago exercise program below). Furthermore, supplementary benefits could also be obtained from functional activity, such as climbing stairs or sitting to standing, flexibility and endurance. The exercises should be tailored for intensity and progressive in intensity. Walking as an exercise on its own was not effective at reducing falls [98–100].

In community-dwelling older people, exercise programs are extremely effective at reducing falls. There are exercise programs, such as Otago [101], that are easily accessible, validated and standardized. The Otago program is suitable for frailer older people at high risk of falls and consists of an individualized program of specific exercises (30 minutes, 3 times per week) to progressively increase lower limb strength (hip, thigh, knee and ankle exercises with an ankle weight) and balance (standing with one foot in front of the other, walking with one foot placed in front of the other, walking on toes and heels, walking backwards, sideways and turning around, stepping over objects, stair climbing, rising from a sitting to a standing position, knee squat) and general physical activity (i.e., walking). The Otago program can be used to initiate people into exercise before moving on to more challenging strength and balance exercises such as those in the PSI/FaME program [102].

In some instances, exercise is effective only amongst a subset of trial participants. For example, Tai Chi, was effective only when performed by people at low risk of falling. Since Tai Chi involves maintaining specific positions to increase balance and muscle strength, this type of exercise could be more challenging for people with poor balance, dizziness and postural hypotension. Differently from Tai Chi, other exercise programs that target muscle strength and balance, such as the Otago and the Falls Management Exercise (FaME) programs, have been demonstrated to reduce falls in older people at higher risk of falling ([102, 103]). These programs were not demonstrated to be effective in participants who had suffered a stroke or had Parkinson’s disease [36, 37].

The impact of exercise, performed as a single intervention, on falls amongst residents in care facilities, was mixed. Silva et al 2013 [51] observed a decline in falls, but combined RCTs of exercise, as a single intervention, with RCTs of multifactorial interventions. In contrast, Cameron et al 2012 [38] and Sherrington et al 2011 [53] combined only trials using exercise as a single intervention and saw no effect on falls in residents of care facilities. Given that multifactorial interventions are composed of various elements, when trials of exercise as a single intervention are combined with multifactorial intervention trials, it is impossible to conclude that only exercise is responsible for the decline in falls. Therefore, based on the results of Cameron et al, exercise alone is unable to reduce falls in residents of care facilities.

Another review (Chan et al 2015 [91]) observed a marked decline in the number of falls in cognitively impaired older adults, which represents the majority of subjects assisted in nursing homes. However, the authors stated that their review was limited by the small number of RCTs and the heterogeneity in the level of cognitive impairment of the participants, settings and dropout rates.

The effect of exercise on subacute wards in hospitals was positive (Cameron et al 2012 [38]) although this was based on very limited data and should be taken with caution.

### Environment/assistive technology

Environmental hazards are a frequent cause of falls in older adults [104]. Environmental intervention consists in assessing home hazards and performing modifications (e.g., employing high levels of illumination and night lights for low and uneven lighting, using non-slip floor surfaces for slippery floors, removing or attaching loose rugs, clearing walkways for obstructed walkways, installing grab rails when absent in the bathroom (shower, bathtub, toilet), using a toilet seat raiser for low toilet seats, installing handrails when not present on stairs, modifying stairs or installing ramps on steep or narrow stairs).

This intervention reduced falls (fall rate and number of fallers) only in individuals with a higher risk of falling (i.e. people with a history of falling or ≥1 risk factors) and when administered by an occupational therapist ([35]). The former is an important result, because this intervention is a relatively straightforward way to prevent falls in a vulnerable older population, while multimorbidity or polypharmacy may be difficult to reduce or eliminate as risk factors for falling. Moreover, it is not surprising that this type of intervention is effective when performed by a qualified professional, given their specific expertise to deal with this type of vulnerable population.

### Multiple interventions

Goodwin et al 2014 [56] pooled various combinations of multiple interventions, based on the recommendation of the US Preventative Services Task Force that a multifactorial approach has only a small benefit to prevent falls in older people [105], and found that both the fall rate, and the number people falling, were reduced. Given the heterogeneity among the multiple intervention trials, combining them in a meta-analysis was not justified. In fact, Gillespie et al 2012 [35] analyzed individual trials of multiple interventions, without performing a meta-analysis. The latter authors stated that few multiple interventions were effective, but that exercise was a component when multiple interventions were effective.

### Multifactorial interventions

Multifactorial interventions, which are based on a strategy of identifying and abating risk factors for falls in each individual, were demonstrated to be effective in fall prevention. This is true in community-dwelling older people as well as in hospitalized older subjects. Since falls are a geriatric syndrome, and therefore usually have a multifactorial etiology, this is not surprising.

On the other hand, in care facilities, there are contrasting results reported by different SRs, which is probably due to the heterogeneity of the trials examining the multifactorial interventions (setting and intervention components). While Cameron et al 2012 [38] did not find a statistically significant reduction in falls (fall rate and number of fallers) in a meta-analysis, Vlaeyen et al 2015 [59] found that the number of falls and recurrent fallers both diminished. One difference between the two SRs was setting. Whereas Cameron et al combined trials of participants requiring intermediate and high levels of assistance, in residential and long-term care facilities, Vlaeyen et al used a precise definition of a nursing home, as part of their inclusion criteria, to select and combine multifactorial intervention trials in a meta-analysis. The influence of the various components of the multifactorial interventions, on the decrease in falls, was investigated by Rimland et al 2015 [18]. By combining only the trials that employed at least exercise, physical environment and assistive technology, in a sensitivity meta-analysis of all the multifactorial intervention trials included in Cameron et al and Valeyen et al, both the fall rate and the number of fallers became statistically significant, and larger, compared to the meta-analysis containing all the multifactorial intervention trials. This result suggested that those three components were crucial for the effectiveness of the multifactorial intervention.

### Other overviews of reviews

During the preparation of this manuscript, and therefore after we performed our systematic search, two umbrella reviews of fall prevention measures in older adults living at home [106], and in long-term care facilities and hospitals [107], were published. Both umbrella reviews reached essentially the same conclusions as the present overview with a few minor differences. First and foremost, the two umbrella reviews examined a total of 26 meta-analyses compared to the 28 systematic reviews with meta-analyses and 31 systematic reviews without meta-analyses, included in the present overview.

The two umbrella reviews only searched for meta-analyses published anytime up to 2014 (August 2014 for fall prevention measures at home and October 2014 for fall prevention in long-term care facilities and hospitals), whereas we searched for systematic reviews and meta-analyses, with a publication time limit from January 2009 to March 2015. In addition, we included fall prevention measures in the 3 settings in a single overview. We did not include vitamin D supplementation as Stubbs et al did, but unlike those authors, we included studies performed in patients with specific diseases. The latter are important, given that older adults often suffer from multimorbidity.

In the present overview, a Delphi method was employed to select the outcomes (critical, important and unimportant), which consists of an international panel of experts who rated outcomes in two rounds, whereas the same method was not used by Stubbs et al. Both umbrella reviews and the present overview evaluated the methodological quality of the systematic reviews and meta-analyses with AMSTAR [21], a systematic review assessment tool.

Regarding the different types of interventions, we agree with the conclusions of Stubbs et al concerning the effect of exercise in community-dwelling adults, and in long-term care facilities (LTCFs). Moreover, we included evidence that exercise in hospital subacute wards was effective (Cameron et al 2012 [30]) although the data were very limited. We disagree with Stubbs et al regarding Silva et al 2013 [51] as an example of recent evidence supporting the effectiveness of exercise. Given that Silva et al 2013 combined RCTs of exercise, as a single intervention, with RCTs of multifactorial interventions, a significant methodological limitation, it cannot be concluded that only exercise is responsible for the decrease in falls.

Regarding environmental interventions in community-dwelling adults, based on the meta-analyses examined, Stubbs et al. stated that there was conflicting evidence. In the present overview, the results of the same type of intervention, reported in the reviews, were more consistent. Furthermore, Gillespie 2012 [35] demonstrated an effect in subjects at high risk of falling, a result not reported by Stubbs et al., and when delivered by an occupational therapist.

Concerning multiple interventions in community-dwelling adults, Stubbs et al., citing the study of Goodwin 2014 [56], commented in the discussion, that “multicomponent interventions (in which the intervention is not specifically tailored to the individual) also can reduce falls is of great interest” [106]. As we discussed above, the meta-analysis of Goodwin 2014 had an important methodological shortcoming, in that various combinations of multiple interventions were combined, but given the heterogeneity among those trials, combining them in a meta-analysis was not justified. Therefore, the assertion of Stubbs et al must be taken with caution.

Finally, in both umbrella reviews, Stubbs et al stated that the quality of evidence was moderate to high, based on the AMSTAR scores of the methodological quality of the meta-analyses. A more appropriate approach to evaluate the evidence is the Grading of Recommendations Assessment, Development, and Evaluation (GRADE) system [108]. We are, in fact, preparing a manuscript of a GRADE evaluation of the evidence supporting the various types of interventions to prevent falls in older people in the three settings.

### Quality of the evidence

Overall, 19% of the reviews (n = 11) were rated as high quality (score 8–11), using the AMSTAR checklist [21]. Reviews with a higher AMSTAR score were more likely to contain more primary studies, to be updated and to perform meta-analysis.

Non-pharmacological interventions are characterized by variation in the type of the intervention used. However, most of the meta-analyses that combined trials with similar interventions showed low heterogeneity, which indicated the robustness of the results.

### Potential limitations

The main limitation of this overview concerned the six-year time restriction, 2009–2015, in which we searched for published systematic reviews. There are many systematic reviews, on non-pharmacological interventions to prevent falls in older people, published each year and systematic reviews published in successive years contain many of the same primary studies. Since we wanted to use the systematic reviews to identify primary studies to formulate clinical questions of prevalent medical conditions affecting older people, which was the main aim of ONTOP, and it was unlikely that we would miss published primary trials, we decided to set this time limit.

The second limitation is that we do not present the data regarding the methodological quality of the primary studies. This issue will be covered in a companion paper where we intend to apply the GRADE approach to the evidence of the efficacy and safety of non-pharmacological interventions for falls prevention.

Another limitation concerns the language of publication, as we included only reviews that were published in English, Italian or Spanish. However, we did not identify any review that was published in Italian or Spanish.

## Conclusions

In this overview of reviews of non-pharmacological interventions to prevent falls in older people in different settings (at home, in care facilities and in hospitals), we evaluated the methodological quality of the systematic reviews, with the AMSTAR tool, in order to help clinicians and healthcare workers to have an easier access to the available scientific evidence. The effectiveness of the different ProFaNE interventions to reduce falls in older people varied. Multifactorial interventions that are tailored to counteract fall risk factors, identified through an individual assessment, was the most consistently effective intervention among various settings. Exercise (group and individual home-based), frequently incorporating balance and strength training, was able to reduce falls in community-dwelling older adults. This same type of intervention had contrasting effects on fall rate and the number of fallers in hospital subacute wards and was ineffective in care facilities. Multiple interventions containing exercise were usually effective. On the other hand, some interventions were effective only in subsets of trial participants, such as Tai Chi in people at low risk of falling, and environmental assessment and modification in people at high risk of falling. Finally, knowledge or educational interventions were ineffective.

## Supporting Information

S1 Fig Search Strategy. (TIF)

S1 Table PRISMA 2009 checklist. (DOC)

S2 Table Excluded studies with reasons. (DOCX)

S3 Table AMSTAR scores. (DOCX)

S4 Table PROFANE interventions. (XLSX)
