# Supplementary figures and images for: Effectiveness of Non-Pharmacological Interventions to Prevent Falls in Older People: A Systematic Overview. The SENATOR Project ONTOP Series
Source: PLoS One. 2016 Aug 25;11(8):e0161579. doi: 10.1371/journal.pone.0161579 (PMC4999091; doi:10.1371/journal.pone.0161579)

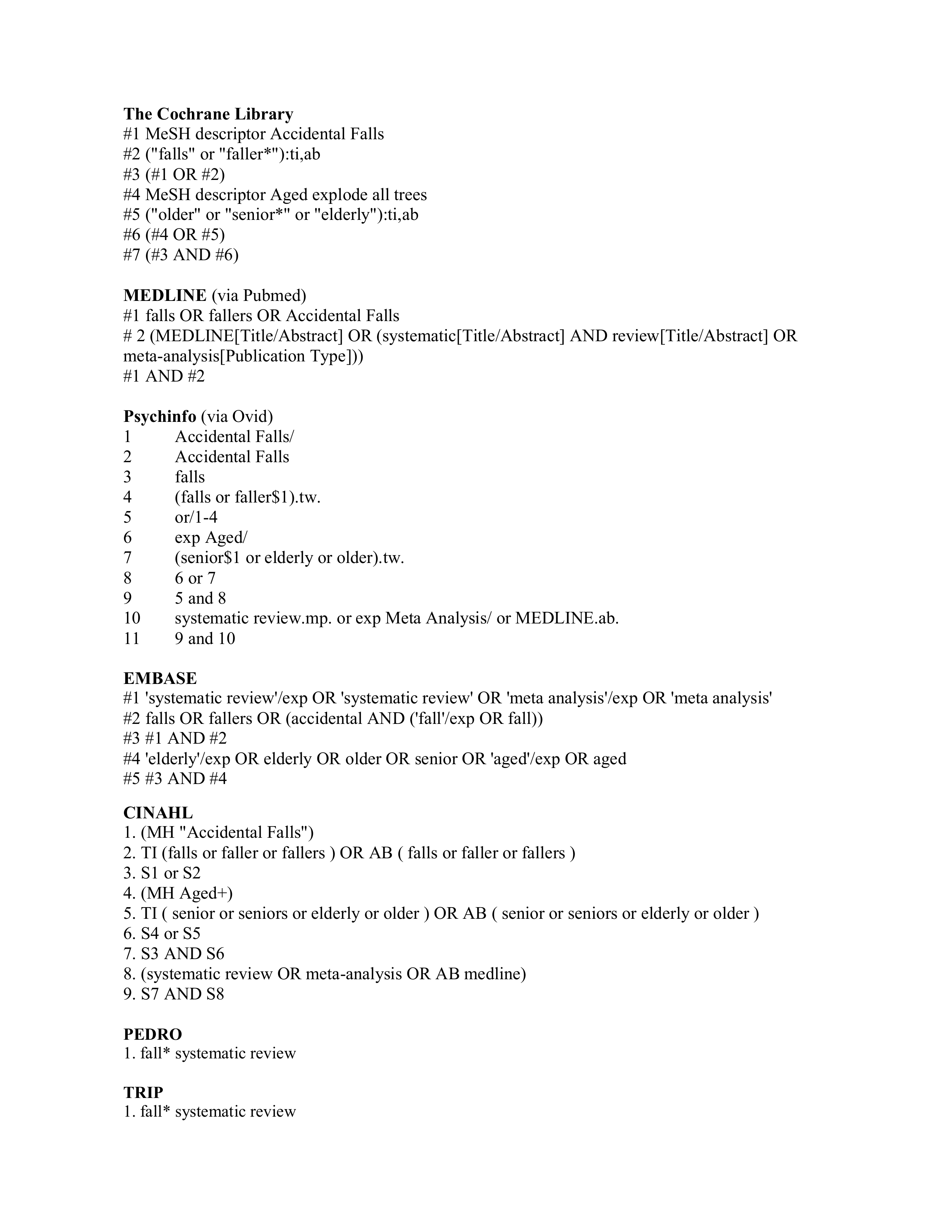

Supplement: S1 Fig — (TIF) [file pone.0161579.s001.tif]
