# Supplementary material for: Effectiveness of Non-Pharmacological Interventions to Prevent Falls in Older People: A Systematic Overview. The SENATOR Project ONTOP Series
Source: PLoS One. 2016 Aug 25;11(8):e0161579. doi: 10.1371/journal.pone.0161579 (PMC4999091; doi:10.1371/journal.pone.0161579)
Supplement: S2 Table — (DOCX) [file pone.0161579.s003.docx]

**S1 Table. Excluded studies**

| **Study** | **Reason for exclusion** |
| --- | --- |
| Abdel-Rahman 2011 ([1](#_ENREF_1)) | Does not include studies of non-pharmacological interventions |
| Anthony 2013 ([2](#_ENREF_2)) | Does not include studies of falls |
| Aromataris 2010 ([3](#_ENREF_3)) | Not a review |
| Beauchet 2011 ([4](#_ENREF_4)) | Guideline |
| Boaro 2009 ([5](#_ENREF_5)) | Commentary of another paper |
| Body 2011 ([6](#_ENREF_6)) | An osteoporosis management review~~s~~ |
| Carpenter 2010 ([7](#_ENREF_7)) | Commentary of another paper |
| Chase 2012 ([8](#_ENREF_8)) | Did not meet one of the inclusion criteria: the use of at least one medical literature electronic database. |
| Child 2012 ([9](#_ENREF_9)) | Does not include studies of falls |
| Church 2011 ([10](#_ENREF_10)) | Economic evaluation of falls prevention programs |
| Cumbler 2011 ([11](#_ENREF_11)) | Did not meet one of the inclusion criteria: the use of at least one medical literature electronic database. |
| Davis 2010 ([12](#_ENREF_12)) | Economic evaluation of falls prevention programs |
| Donaldson 2009 ([13](#_ENREF_13)) | Does not include studies of non-pharmacological interventions |
| Dukyoo 2009 ([14](#_ENREF_14)) | Does not include studies of falls |
| Ehrlich 2009 ([15](#_ENREF_15)) | Not a review |
| Fairhall 2011 ([16](#_ENREF_16)) | Does not include studies of falls |
| Fox 2013 ([17](#_ENREF_17)) | Descriptive systematic review of Fox 2012 |
| Frost 2012 ([18](#_ENREF_18)) | Not a review |
| Giangregorio 2013 ([19](#_ENREF_19)) | Does not include studies of falls |
| Gillespie 2009 ([20](#_ENREF_20)) | Review withdrawn |
| Goodwin 2011 ([21](#_ENREF_21)) | A review of methods to implement fall prevention programs |
| Grabiner 2014 ([22](#_ENREF_22)) | Commentary of another paper |
| Granacher 2009 ([23](#_ENREF_23)) | Does not contain any RCTs |
| Haines 2011 ([24](#_ENREF_24)) | Not a review |
| Handoll 2010 ([25](#_ENREF_25)) | Commentary of another paper |
| Healey 2008 ([26](#_ENREF_26)) | Does not contain any RCTs |
| Healey 2009 ([27](#_ENREF_27)) | Duplicate of Healey 2008 |
| Healey 2012 ([28](#_ENREF_28)) | Not a review |
| Holt 2012 ([29](#_ENREF_29)) | Falls were not reported |
| Howe 2011 ([30](#_ENREF_30)) | Does not include studies of falls |
| Jensen 2011 ([31](#_ENREF_31)) | Did not meet one of the inclusion criteria: the use of at least one medical literature electronic database. ~~reviews~~ |
| Jeter 2014 ([32](#_ENREF_32)) | Does not include studies of falls |
| Jung 2009 ([33](#_ENREF_33)) | Does not include studies of falls |
| Kiel 2011 ([34](#_ENREF_34)) | Commentary of another paper |
| Laufer 2014 ([35](#_ENREF_35)) | Falls were not reported |
| Maciaszek 2010 ([36](#_ENREF_36)) | Does not include studies of falls |
| Mat 2015 ([37](#_ENREF_37)) | Does not include studies of falls |
| Naqvi 2009 ([38](#_ENREF_38)) | Not a review |
| Oliver 2010 ([39](#_ENREF_39)) | Did not meet one of the inclusion criteria: the use of at least one medical literature electronic database. |
| Paton 2014 ([40](#_ENREF_40)) | Protocol for a systematic review |
| Schwickert 2013 ([41](#_ENREF_41)) | Did not include fall prevention interventions |
| Sze 2012 ([42](#_ENREF_42)) | Protocol for a systematic review |
| Tinetti 2013 ([43](#_ENREF_43)) | Not a review |
| Tomlinson 2013 ([44](#_ENREF_44)) | Falls were not reported |
| Trombetti 2009 ([45](#_ENREF_45)) | Published in French |
| Vaapio 2009 ([46](#_ENREF_46)) | Does not include studies of falls |
| van Hateren 2011 ([47](#_ENREF_47)) | Not a review |
| Visschedijk 2010 ([48](#_ENREF_48)) | Fear of falling review |
| Wu 2010 ([49](#_ENREF_49)) | Cost-effectiveness analysis of a proposed falls prevention program |

1. Abdel-Rahman EM, Turgut F, Turkmen K, Balogun RA. Falls in elderly hemodialysis patients. Qjm. 2011;104(10):829-38. PubMed PMID: 2011535308 MEDLINE PMID 21750022 (<http://www.ncbi.nlm.nih.gov/pubmed/21750022>) FULL TEXT LINK <http://dx.doi.org/10.1093/qjmed/hcr108>. English.

2. Anthony K, Robinson K, Logan P, Gordon AL, Harwood RH, Masud T. Chair-Based Exercises for Frail Older People: A Systematic Review. BioMed research international. 2013;2013:309506. PubMed PMID: 24089670. Pubmed Central PMCID: PMC3782120. Epub 2013/10/04. Eng.

3. Aromataris E. Review summaries: evidence for nursing practice. Interventions to reduce the incidence of falls in older adult patients in acute care hospitals. Journal of Advanced Nursing. 2010;66(6):1209-11. PubMed PMID: 2010651890. Language: English. Entry Date: 20100618. Revision Date: 20100618. Publication Type: journal article.

4. Beauchet O, Dubost V, Revel Delhom C, Berrut G, Belmin J. How to manage recurrent falls in clinical practice: guidelines of the French Society of Geriatrics and Gerontology. The journal of nutrition, health & aging. 2011 Jan;15(1):79-84. PubMed PMID: 21267524. Epub 2011/01/27. eng.

5. Boaro N. Review: Exercise programmes prevent falls in elderly people. Evidence Based Nursing. 2009;12(3):86-. PubMed PMID: 2010325962. Language: English. Entry Date: 20090925. Revision Date: 20130524. Publication Type: journal article.

6. Body JJ, Bergmann P, Boonen S, Boutsen Y, Bruyere O, Devogelaer JP, et al. Non-pharmacological management of osteoporosis: a consensus of the Belgian Bone Club. Osteoporosis international : a journal established as result of cooperation between the European Foundation for Osteoporosis and the National Osteoporosis Foundation of the USA. 2011 Nov;22(11):2769-88. PubMed PMID: 21360219. Pubmed Central PMCID: PMC3186889. Epub 2011/03/02. eng.

7. Carpenter CR. Preventing Falls in Community-Dwelling Older Adults. Annals of Emergency Medicine. 2010 March;55(3):296-8. PubMed PMID: 2010105189 MEDLINE PMID 19615786 (<http://www.ncbi.nlm.nih.gov/pubmed/19615786>) FULL TEXT LINK <http://dx.doi.org/10.1016/j.annemergmed.2009.06.014>. English.

8. Chase CA, Mann K, Wasek S, Arbesman M. Systematic review of the effect of home modification and fall prevention programs on falls and the performance of community-dwelling older adults. The American journal of occupational therapy : official publication of the American Occupational Therapy Association. 2012 May-Jun;66(3):284-91. PubMed PMID: 22549593. Epub 2012/05/03. eng.

9. Child S, Goodwin V, Garside R, Jones-Hughes T, Boddy K, Stein K. Factors influencing the implementation of fall-prevention programmes: a systematic review and synthesis of qualitative studies. Implementation science : IS. 2012;7:91. PubMed PMID: 22978693. Pubmed Central PMCID: PMC3576261. Epub 2012/09/18. eng.

10. Church J, Goodall S, Norman R, Haas M. An economic evaluation of community and residential aged care falls prevention strategies in NSW. New South Wales public health bulletin. 2011 Jun;22(3-4):60-8. PubMed PMID: 21632001. Epub 2011/06/03. eng.

11. Cumbler E, Likosky D. In-hospital falls: evaluation and response. Continuum (Minneapolis, Minn). 2011 Oct;17(5 Neurologic Consultation in the Hospital):1063-76. PubMed PMID: 22809982. Epub 2012/07/20. eng.

12. Davis JC, Robertson MC, Ashe MC, Liu-Ambrose T, Khan KM, Marra CA. Does a home-based strength and balance programme in people aged >or =80 years provide the best value for money to prevent falls? A systematic review of economic evaluations of falls prevention interventions. British journal of sports medicine. 2010;44(2):80-9. PubMed PMID: 2010564798. Language: English. Entry Date: 20110311. Revision Date: 20110520. Publication Type: journal article.

13. Donaldson MG, Sobolev B, Cook WL, Janssen PA, Khan KM. Analysis of recurrent events: a systematic review of randomised controlled trials of interventions to prevent falls. Age Ageing. 2009 Mar;38(2):151-5. PubMed PMID: 19106254. Epub 2008/12/25. eng.

14. Dukyoo J, Juhee L, Lee SM. A meta-analysis of fear of falling treatment programs for the elderly. West J Nurs Res. 2009 Feb;31(1):6-16. PubMed PMID: 18667626. Epub 2008/08/01. eng.

15. Ehrlich A. Evidence-based medicine. Interventions may reduce falls in community-dwelling older adults. Clinical Advisor for Nurse Practitioners. 2009;12(8):67-. PubMed PMID: 2010403509. Language: English. Entry Date: 20091009. Revision Date: 20121109. Publication Type: journal article.

16. Fairhall N, Sherrington C, Clemson L, Cameron ID. Do exercise interventions designed to prevent falls affect participation in life roles? A systematic review and meta-analysis. Age Ageing. 2011 Nov;40(6):666-74. PubMed PMID: 21764816. Epub 2011/07/19. eng.

17. Fox MT, Sidani S, Persaud M, Tregunno D, Maimets I, Brooks D, et al. Acute care for elders components of acute geriatric unit care: systematic descriptive review. J Am Geriatr Soc. 2013 Jun;61(6):939-46. PubMed PMID: 23692509. Epub 2013/05/23. eng.

18. Frost H, Haw S, Frank J. Interventions in community settings that prevent or delay disablement in later life: an overview of the evidence. Quality in Ageing & Older Adults. 2012;13(3):212-30. PubMed PMID: 2011749812. Language: English. Entry Date: 20121130. Revision Date: 20130104. Publication Type: journal article.

19. Giangregorio LM, Macintyre NJ, Thabane L, Skidmore CJ, Papaioannou A. Exercise for improving outcomes after osteoporotic vertebral fracture. The Cochrane database of systematic reviews. 2013;1:CD008618. PubMed PMID: 23440829. Epub 2013/02/27. eng.

20. Gillespie LD, Robertson MC, Gillespie WJ, Lamb SE, Gates S, Cumming RG, et al. Interventions for preventing falls in older people living in the community. The Cochrane database of systematic reviews. 2009 (2):CD007146. PubMed PMID: 19370674. Epub 2009/04/17. eng.

21. Goodwin V, Jones-Hughes T, Thompson-Coon J, Boddy K, Stein K. Implementing the evidence for preventing falls among community-dwelling older people: a systematic review. Journal of safety research. 2011 Dec;42(6):443-51. PubMed PMID: 22152262. Epub 2011/12/14. eng.

22. Grabiner MD. Exercise-based fall prevention programmes decrease fall-related injuries. Evidence Based Nursing. 2014 February 11, 2014.

23. Granacher U, Gollhofer A, Hortobagyi T, Kressig RW, Muehlbauer T. The importance of trunk muscle strength for balance, functional performance, and fall prevention in seniors: a systematic review. Sports medicine (Auckland, NZ). 2013 Jul;43(7):627-41. PubMed PMID: 23568373. Epub 2013/04/10. eng.

24. Haines TP, Hill AM. Inconsistent results in meta-analyses for the prevention of falls are found between study-level data and patient-level data. Journal of clinical epidemiology. 2011 Feb;64(2):154-62. PubMed PMID: 20947297. Epub 2010/10/16. eng.

25. Handoll H. Prevention of falls and fall related injuries in older people in nursing homes and hospitals. Injury Prevention. 2010 2010;16(2):137-8. English.

26. Healey F, Oliver D, Milne A, Connelly JB. The effect of bedrails on falls and injury: a systematic review of clinical studies. Age Ageing. 2008 Jul;37(4):368-78. PubMed PMID: 18495686. Epub 2008/05/23. eng.

27. Healey F, Oliver D. Bedrails, falls and injury: evidence or opinion? A review of their use and effects. Nursing times. 2009 Jul 7-13;105(26):20-4. PubMed PMID: 19736820. Epub 2009/09/10. eng.

28. Healey F, Darowski A. Older patients and falls in hospital. Clinical Risk. 2012;18(5):170-6. PubMed PMID: 2011746553. Language: English. Entry Date: 20121123. Revision Date: 20130927. Publication Type: journal article.

29. Holt KR, Haavik H, Elley CR. The effects of manual therapy on balance and falls: a systematic review. J Manipulative Physiol Ther. 2012 Mar-Apr;35(3):227-34. PubMed PMID: 22343006. Epub 2012/02/22. eng.

30. Howe TE, Shea B, Dawson LJ, Downie F, Murray A, Ross C, et al. Exercise for preventing and treating osteoporosis in postmenopausal women. The Cochrane database of systematic reviews. 2011 (7):CD000333. PubMed PMID: 21735380. Epub 2011/07/08. eng.

31. Jensen LE, Padilla R. Effectiveness of interventions to prevent falls in people with Alzheimer's disease and related dementias. The American journal of occupational therapy : official publication of the American Occupational Therapy Association. 2011 Sep-Oct;65(5):532-40. PubMed PMID: 22026321. Epub 2011/10/27. eng.

32. Jeter PE, Nkodo AF, Moonaz SH, Dagnelie G. A systematic review of yoga for balance in a healthy population. Journal of alternative and complementary medicine (New York, NY). 2014 Apr;20(4):221-32. PubMed PMID: 24517304. Pubmed Central PMCID: PMC3995122. Epub 2014/02/13. eng.

33. Jung D, Lee J, Lee SM. A meta-analysis of fear of falling treatment programs for the elderly (Structured abstract). Western Journal of Nursing Research [Internet]. 2009; 31(1):[6-16 pp.]. Available from: <http://onlinelibrary.wiley.com/o/cochrane/cldare/articles/DARE-12009104508/frame.html>.

34. Kiel DP. Review: Exercise/physical therapy and vitamin D each reduce risk for falls in older community-dwelling adults. ACP Journal Club. 2011;154(4):4-. PubMed PMID: 2011246346. Language: English. Entry Date: 20110909. Revision Date: 20110909. Publication Type: journal article.

35. Laufer Y, Dar G, Kodesh E. Does a Wii-based exercise program enhance balance control of independently functioning older adults? A systematic review. Clinical interventions in aging. 2014;9:1803-13. PubMed PMID: 25364238. Pubmed Central PMCID: PMC4211857. Epub 2014/11/05. eng.

36. Maciaszek J, Osinski W. The effects of Tai Chi on body balance in elderly people -- a review of studies from the early 21st century. American Journal of Chinese Medicine. 2010;38(2):219-29. PubMed PMID: 2010648324. Language: English. Entry Date: 20100611. Revision Date: 20100716. Publication Type: journal article.

37. Mat S, Tan MP, Kamaruzzaman SB, Ng CT. Physical therapies for improving balance and reducing falls risk in osteoarthritis of the knee: a systematic review. Age Ageing. 2015 Jan;44(1):16-24. PubMed PMID: 25149678. Epub 2014/08/26. eng.

38. Naqvi F, Lee S, Fields SD. Appraising a guideline for preventing acute care falls. Geriatrics. 2009 2009;64(3):13+26. PubMed PMID: 2010446478. English.

39. Oliver D, Healey F, Haines TP. Preventing falls and fall-related injuries in Hospitals. Clinics in Geriatric Medicine. 2010 November;26(4):645-92. PubMed PMID: 2010562022 MEDLINE PMID 20934615 (<http://www.ncbi.nlm.nih.gov/pubmed/20934615>) FULL TEXT LINK <http://dx.doi.org/10.1016/j.cger.2010.06.005>. English.

40. Paton J, Collings R, Glasser S, Kent B. The effects of foot and ankle devices on balance, gait and falls in adults with sensory perception loss: a systematic review protocol. JBI Database of Systematic Reviews & Implementation Reports. 2014;12(11):74-91.

41. Schwickert L, Becker C, Lindemann U, Marechal C, Bourke A, Chiari L, et al. Fall detection with body-worn sensors : a systematic review. Zeitschrift fur Gerontologie und Geriatrie. 2013 Dec;46(8):706-19. PubMed PMID: 24271251. Epub 2013/11/26. eng.

42. Sze TW, Leng CY, Lin SKS. The effectiveness of physical restraints in reducing falls among adults in acute care hospitals and nursing homes: A systematic review. JBI Database of Systematic Reviews and Implementation Reports. 2012 2012;10(5):307-51. PubMed PMID: 2013554654. English.

43. Tinetti M. 2012 - Review: Acute geriatric unit care reduces falls, delirium, and functional decline. ACP Journal Club. 2013;158(12):1-. PubMed PMID: 2012162230. Language: English. Entry Date: 20130809. Revision Date: 20130809. Publication Type: journal article.

44. Tomlinson CL, Patel S, Meek C, Herd CP, Clarke CE, Stowe R, et al. Physiotherapy versus placebo or no intervention in Parkinson's disease. The Cochrane database of systematic reviews. 2013;9:CD002817. PubMed PMID: 24018704. Epub 2013/09/11. eng.

45. Trombetti A, Hars M, Marcant D, Rizzoli R, Ferrari S. Fall prevention: A challenge in the strategy of fracture prevention in the elderly ORIGINAL (NON-ENGLISH) TITLE Prevention de la chute: Un enjeu de taille dans la strategie visant a prevenir les fractures chez le sujet age. Revue Medicale Suisse. 2009 Juin;5(207):1318-24. PubMed PMID: 2009331463 MEDLINE PMID 19626932 (<http://www.ncbi.nlm.nih.gov/pubmed/19626932)>. French.

46. Vaapio SS, Salminen MJ, Ojanlatva A, Kivela SL. Quality of life as an outcome of fall prevention interventions among the aged: a systematic review. European journal of public health. 2009 Jan;19(1):7-15. PubMed PMID: 18971207. Epub 2008/10/31. eng.

47. van Hateren KJ, Kamper AM, Bilo HJ. Lack of evaluation of the effects of single preventive measures for falling... Arch Intern Med. 2010 Jul 12;170(13):1110-7. Archives of Internal Medicine. 2011;171(2):181-2. PubMed PMID: 2010920176. Language: English. Entry Date: 20110401. Revision Date: 20120302. Publication Type: journal article.

48. Visschedijk J, Achterberg W, Van Balen R, Hertogh C. Fear of falling after hip fracture: a systematic review of measurement instruments, prevalence, interventions, and related factors. J Am Geriatr Soc. 2010 Sep;58(9):1739-48. PubMed PMID: 20863333. Epub 2010/09/25. eng.

49. Wu S, Keeler EB, Rubenstein LZ, Maglione MA, Shekelle PG. A cost-effectiveness analysis of a proposed national falls prevention program. Clin Geriatr Med. 2010 Nov;26(4):751-66. PubMed PMID: 20934620. Epub 2010/10/12. eng.
