# Supplementary material for: Effectiveness of Non-Pharmacological Interventions to Prevent Falls in Older People: A Systematic Overview. The SENATOR Project ONTOP Series
Source: PLoS One. 2016 Aug 25;11(8):e0161579. doi: 10.1371/journal.pone.0161579 (PMC4999091; doi:10.1371/journal.pone.0161579)
Supplement: S3 Table — (DOCX) [file pone.0161579.s004.docx]

**S2 Table:** Methodological Quality Assessment of the included studies Systematic Reviews according to the AMSTAR criteria

|  |  | **AMSTAR Items** | | | | | | | | | | | |
| --- | --- | --- | --- | --- | --- | --- | --- | --- | --- | --- | --- | --- | --- |
|  |  | **1** | **2** | **3** | **4** | **5** | **6** | **7** | **8** | **9** | **10** | **11** | **Rating** |
| Al-Aama 2011 | | No | No | No | No | No | No | No | NA | NA | No | No | 0 |
| Allen 2011 | | No | Yes | Yes | No | Yes | Yes | Yes | Yes | Yes | Yes | No | 8 |
| Anderson 2011 | | Yes | Yes | Yes | Yes | Yes | Yes | Yes | Yes | Yes | No | No | 10 |
| Anderson 2012 | | Yes | Yes | Yes | Yes | Yes | Yes | Yes | Yes | Yes | No | No | 9 |
| Batchelor 2010 | | CA | No | Yes | No | No | Yes | Yes | Yes | Yes | No | No | 5 |
| Batchelor 2013 | | No | Yes | Yes | No | No | Yes | Yes | Yes | Yes | No | No | 6 |
| Bunn 2014 | | Yes | Yes | Yes | No | No | Yes | Yes | No | Yes | No | No | 6 |
| Burton 2015 | | No | CA | Yes | No | No | Yes | Yes | Yes | Yes | Yes | No | 6 |
| Cadore 2013 | | CA | Yes | Yes | No | No | Yes | Yes | No | NA | No | No | 4 |
| Cameron 2010 | | Yes | Yes | Yes | No | Yes | Yes | Yes | Yes | Yes | No | No | 8 |
| Cameron 2012 | | Yes | Yes | Yes | No | Yes | Yes | Yes | Yes | Yes | No | No | 8 |
| Chan 2015 | | No | Yes | Yes | No | No | Yes | Yes | No | Yes | Yes | No | 6 |
| Choi 2011 | | No | No | Yes | No | No | Yes | Yes | No | NA | Yes | No | 4 |
| Choi 2012 | | No | Yes | Yes | No | No | Yes | Yes | Yes | Yes | Yes | No | 7 |
| Combes 2013 | | No | Yes | Yes | No | Yes | No | Yes | Yes | NA | No | No | 5 |
| Corrieri 2011 | | No | CA | No | No | No | Yes | No | No | NA | No | No | 1 |
| de Kam 2009 | | No | Yes | Yes | No | No | Yes | Yes | No | NA | No | No | 4 |
| Desapriya 2010 | | No | Yes | Yes | Yes | No | Yes | Yes | Yes | Yes | No | No | 7 |
| DiBardino 2012 | | No | CA | Yes | No | No | Yes | Yes | Yes | Yes | Yes | No | 6 |
| El-Khoury 2013 | | No | CA | Yes | No | No | Yes | Yes | No | Yes | Yes | No | 5 |
| Fox 2012 | | No | Yes | Yes | No | No | Yes | Yes | Yes | Yes | Yes | No | 7 |
| Gillespie 2010 | | Yes | Yes | Yes | Yes | Yes | Yes | Yes | Yes | Yes | Yes | No | 10 |
| Gillespie 2012 | | Yes | Yes | Yes | Yes | Yes | Yes | Yes | Yes | Yes | Yes | No | 10 |
| Gleeson 2014 | | No | Yes | Yes | No | No | Yes | Yes | Yes | Yes | No | No | 6 |
| Goodwin 2014 | | Yes | Yes | Yes | No | No | Yes | Yes | Yes | Yes | Yes | No | 8 |
| Gregory 2009 | | No | Yes | Yes | No | No | Yes | Yes | No | NA | No | No | 4 |
| Gschwind 2011 | | No | Yes | Yes | No | No | Yes | Yes | Yes | Yes | No | No | 6 |
| Guo 2014 | | No | No | Yes | No | No | CA | No | No | No | No | No | 1 |
| Hempel 2013 | | No | No | Yes | No | No | Yes | No | No | Yes | Yes | No | 4 |
| Ishigaki 2014 | | No | Yes | Yes | No | No | Yes | Yes | Yes | Yes | No | No | 6 |
| Ishikawa 2013 | | No | CA | Yes | No | Yes | Yes | Yes | Yes | NA | No | No | 5 |
| Kosse 2013 | | No | CA | Yes | No | No | Yes | Yes | Yes | NA | No | No | 4 |
| Laguna-Parras 2010 | | No | CA | Yes | No | Yes | Yes | No | NA | NA | No | No | 3 |
| Lam 2012 | | No | CA | Yes | No | No | Yes | Yes | Yes | NA | Yes | No | 5 |
| Lee 2014 | | No | No | Yes | No | Yes | Yes | Yes | Yes | Yes | No | No | 6 |
| Leung 2011 | | No | CA | Yes | No | No | Yes | Yes | No | Yes | No | No | 4 |
| Logghe 2010 | | No | Yes | Yes | No | No | Yes | Yes | No | Yes | Yes | No | 6 |
| Low 2009 | | No | CA | Yes | No | No | Yes | Yes | Yes | Yes | No | No | 5 |
| Martin 2013 | | No | CA | No | No | No | Yes | Yes | No | Yes | No | No | 3 |
| McMahon 2012 | | No | Yes | Yes | No | No | Yes | No | No | NA | No | No | 3 |
| Miake-Lye 2013 | | No | CA | Yes | No | No | Yes | No | NA | NA | No | No | 2 |
| Michael 2010 | | No | Yes | Yes | No | Yes | Yes | Yes | No | Yes | No | No | 6 |
| Neyens 2010 | | No | Yes | Yes | No | No | Yes | No | NA | Yes | No | No | 4 |
| Petridou 2009 | | No | CA | Yes | No | No | Yes | No | NA | Yes | Yes | No | 4 |
| Santesso 2014 | | Yes | Yes | Yes | Yes | Yes | Yes | Yes | Yes | Yes | Yes | No | 10 |
| Schleicher 2012 | | No | CA | Yes | No | No | Yes | No | NA | NA | No | No | 2 |
| Schoene 2014 | | No | CA | Yes | No | No | Yes | Yes | Yes | Yes | No | No | 5 |
| Sherrington 2011* | | No | Yes | Yes | No | No | Yes | Yes | No | No | Yes | No | 5 |
| Silva 2013 | | No | CA | Yes | No | No | Yes | Yes | Yes | Yes | No | No | 5 |
| Simek 2012 | | No | CA | Yes | No | No | Yes | Yes | Yes | No | No | No | 4 |
| Simpson 2013 | | No | CA | No | No | No | Yes | No | NA | NA | No | No | 1 |
| Sitjà-Rabert 2012 | | No | Yes | Yes | No | No | Yes | Yes | Yes | NA | No | No | 5 |
| Thomas 2010 | | No | CA | Yes | Yes | No | Yes | Yes | Yes | Yes | No | No | 6 |
| Verheyden 2013 | | Yes | Yes | Yes | Yes | Yes | Yes | Yes | Yes | Yes | No | No | 9 |
| Vlaeyen 2015 | | Yes | Yes | Yes | No | No | Yes | Yes | Yes | Yes | Yes | No | 8 |
| Voigt-Radloff 2013 | | No | Yes | No | No | Yes | Yes | Yes | No | Yes | No | No | 5 |
| Wallis 2011 | | No | CA | No | No | No | No | No | NA | NA | No | No | 0 |
| Winter 2013 | | No | Yes | Yes | No | No | Yes | Yes | No | Yes | No | No | 5 |
| Wooton 2010 | | No | No | Yes | No | No | Yes | No | NA | NA | No | No | 2 |

*The AMSTAR score of Sherrington 2011 was based on the AMSTAR evaluation of Sherrington 2008.

All 11-items were scored as “Yes”, “No”, “Can’t Answer” or “Not Applicable”. AMSTAR comprises the following items:

1. ‘a priori’ design provided;

2. duplicate study selection/data extraction;

3. comprehensive literature search;

4. status of publication as inclusion criteria (i.e., grey or unpublished literature);

5. list of studies included/excluded provided;

6. characteristics of included studies documented;

7. scientific quality assessed and documented;

8. appropriate formulation of conclusions (based on methodological rigor and scientific quality of the studies);

9. appropriate methods of combining studies (homogeneity test, effect model used and sensitivity analysis);

10. assessment of publication bias (graphic and/or statistical test);

11. conflict of interest statement.
